# Association of triglyceride glucose-related parameters with all-cause mortality and cardiovascular disease in NAFLD patients: NHANES 1999–2018

**DOI:** 10.1186/s12933-024-02354-4

**Published:** 2024-07-18

**Authors:** Yusha Zhang, Fengjiao Wang, Jianjun Tang, Li Shen, Jia He, Yaqin Chen

**Affiliations:** Department of Cardiology, The Second Xiangya Hospital, Central South University, Hunan Province, Changsha, 410011 Hunan China

**Keywords:** Triglyceride-glucose index, Triglyceride glucose-waist height ratio, NAFLD, All-cause mortality, Cardiovascular disease, NHANES

## Abstract

**Background:**

The relationship between the triglyceride-glucose (TyG) index and its derived index, the triglyceride glucose-waist height ratio (TyG-WHtR), with mortality and cardiovascular diseases (CVDs) in patients with non-alcoholic fatty liver disease (NAFLD) remains unclear.

**Methods:**

This study enrolled 6627 adults aged 18 and above diagnosed NAFLD from the National Health and Nutrition Examination Survey (NHANES, 1999–2018). Binary weighted logistic regression analyses, cox proportional hazards model and restricted cubic spline (RCS) were used to analyze the relationship between TyG and TyG-WHtR with all-cause mortality, CVD mortality and CVDs. Mediation analysis explored the mediating role of glycohemoglobin, insulin and hypertension in the above relationships. Meanwhile, the incremental predictive value of the TyG index and TyG-WHtR was further assessed.

**Results:**

Except for no significant association between the TyG index and both all-cause mortality and chronic heart failure (CHF), both TyG and TyG-WHtR exhibited significant positive correlations or trends of positive correlation with all-cause mortality, CVD mortality, total-CVD, CHF, coronary heart disease (CHD) and angina pectoris. For all-cause mortality, CVD mortality and CHF, TyG-WHtR was a better predictor than TyG (TyG-WHtR: HR 1.31, 95%CI 1.03–1.66; HR 2.22, 95%CI 1.42–3.47; OR 3.99, 95%CI 1.79–8.93). In contrast, TyG index demonstrated a stronger association with total-CVD, CHD and angina pectoris (TyG index: OR 2.00, 95%CI 1.26–3.18; OR 1.85, 95%CI 1.19–2.91; OR 2.93, 95%CI 1.23-7.00). RCS analysis showed that after adjusting for covariates, most of the aforementioned relationships were linear(P overall < 0.0001, P-nonlinear > 0.05), while the associations of the TyG index and TyG-WHtR with all-cause mortality and CHF were non-linear(P overall < 0.0001, P nonlinear < 0.05). The addition of the TyG index and TyG-WHtR to the basic model for outcomes improved the C-statistics, net reclassification improvement value, and integrated discrimination improvement value.

**Conclusions:**

The predictive value of TyG or TyG-WHtR for all-cause mortality and cardiovascular risk in NAFLD patients was significant. The TyG index and TyG-WHtR might be valid predictors of cardiovascular outcomes of patients with NAFLD.

**Supplementary Information:**

The online version contains supplementary material available at 10.1186/s12933-024-02354-4.

## Introduction

Non-alcoholic fatty liver disease (NAFLD) is a systemic metabolic disorder characterized by excessive accumulation of fat in the liver, insulin resistance (IR), and systemic inflammation [1]. Most studies have demonstrated a robust correlation between NAFLD and cardiovascular disease (CVD) [2]. NAFLD is closely associated with an increased risk of major cardiovascular events and cardiac complications, independent of traditional cardiovascular risk factors [3]. Nowadays, numerous models have been developed to predict the risk of CVD in individuals with NAFLD. However, the practicability of most prediction models remains to be confirmed, given the absence of external validation and simulated impact studies [4]. Therefore, reliable indicators for predicting mortality risk and CVD in NAFLD patients are still lacking in clinical practice.

IR refers to the reduced sensitivity of target tissues to normal circulating levels of insulin. This results in ineffective glucose transport into target cells, which in turn leads to the development of metabolic abnormalities such as hyperglycemia [5]. IR is associated with the onset and prognosis of diverse CVDs. A 12-year follow-up cohort study demonstrated a positive correlation between fasting insulin levels and adverse echocardiographic characteristics, as well as an increased risk of heart failure (HF) in patients who had not previously suffered from myocardial infarction or HF [6].

The triglyceride-glucose (TyG) index, derived from the levels of triglycerides (TG) and fasting blood glucose (FBG), serves as a valuable assessment tool for evaluating insulin sensitivity. Although the hyperinsulinemic-euglycemic clamp test remains the gold standard for measuring IR, TyG index have exhibited higher sensitivity and specificity in identifying IR [7, 8]. Moreover, recent studies have highlighted the importance of TyG index as a biomarker for predicting the risk and prognosis of various CVD, including decompensated HF, stroke, and coronary artery disease [9–11].

Obesity is frequently accompanied by hypertension and abnormal lipid metabolism, which significantly increases the risk of developing CVD. Additionally, central obesity can serve as a key indicator for assessing the risk of various obesity-related chronic diseases [12–14]. The indicators for measuring central obesity include waist circumference (WC), waist-to-hip ratio (WHR), and waist-to-height ratio (WHtR). However, since WC can not reflect internal fat distribution and measuring hip circumference for calculating WHR is often challenging, WHtR offers a more direct and straightforward method for assessing central obesity and internal fat content [15]. Researches indicate that TyG and TyG-WHtR are superior to other TyG-related parameters in predicting the risk of NAFLD in general populations [16, 17]. And TyG-WHtR was superior to the TyG index alone in identifying the risk of early diabetes [18, 19] and predicting CVD-related mortality [5]. However, there is currently a deficiency in comparative studies on the correlation between TyG and TyG-obesity index with CVD in patients with NAFLD. This study aims to analyze the association between the TyG index and its combined obesity index, TyG-WHtR, and all-cause mortality, CVD mortality, total CVD, congestive heart failure (CHF), coronary heart disease (CHD) and angina pectoris.

## Method

### Study population

National Health and Nutrition Examination Survey (NHANES) is a national survey of children and adults in the United States. Data of NHANES were collected through personal structured interviews at home, health examinations at a mobile examination center, and specimen analyses in the laboratory (https://www.cdc.gov/nchs/nhanes/index.html*).* NHANES was conducted with approval by the National Center for Health Statistics Ethics Review Board, and obtained informed written consent from all the individuals involved in the study.

**Fig. 1:**
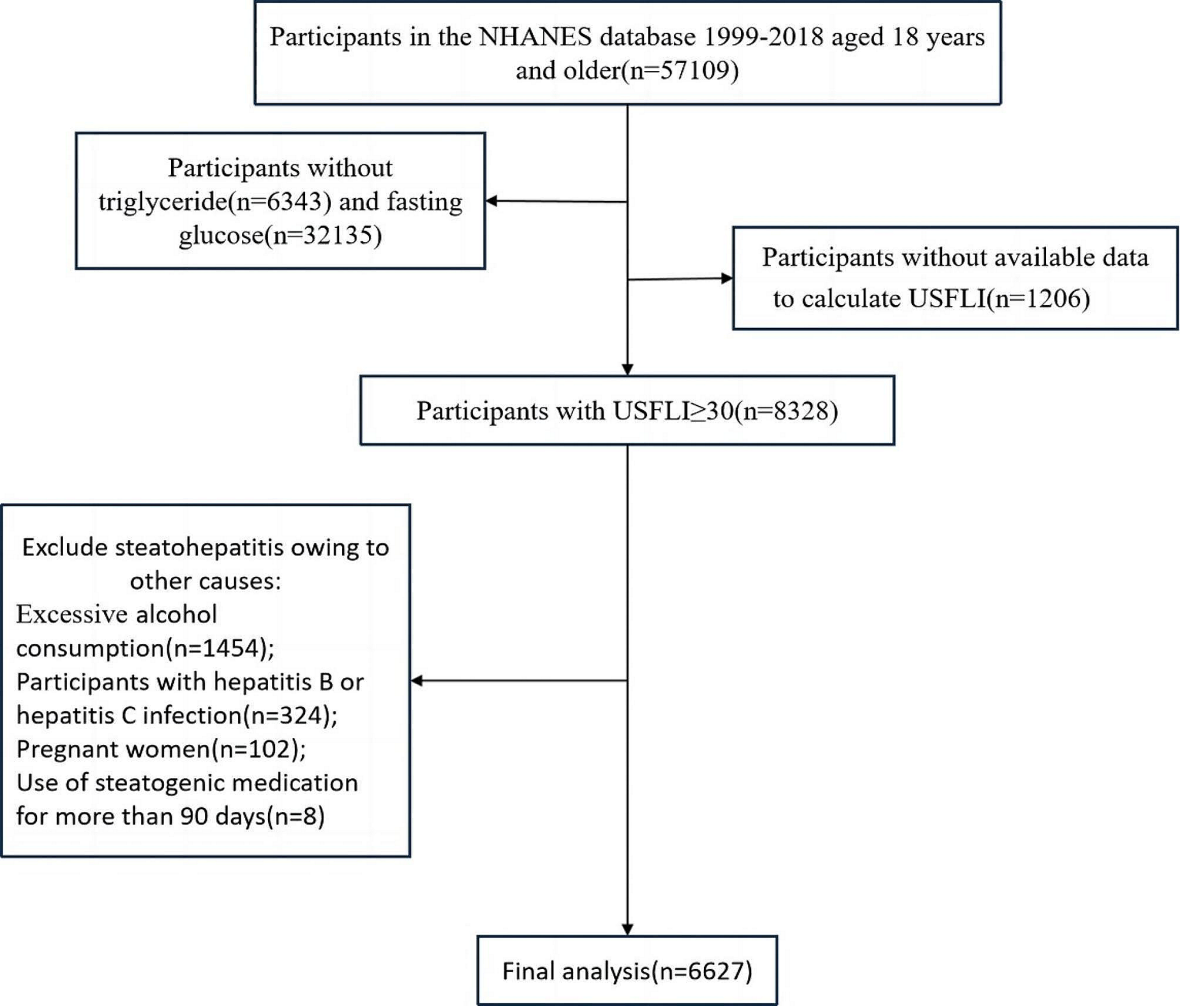
Flowchart depicting the selection of participants

We enrolled adult participants aged 18 years and older with NAFLD who underwent mobile examinations from NHANES 1999–2018, which provided comprehensive data for blood and physical measurements. NAFLD was defined by a US fatty liver index (USFLI) of 30 or higher, a well-established definition with an area under the receiver operating characteristic curve (AUROC) of 0.80 (95% CI: 0.77–0.83) in predicting ultrasound-confirmed NAFLD [20]. The following groups of patients were excluded from the study: (1) excessive alcohol consumption (> 3 drinks per day for the male and > 2 drinks per day for the female); (2) participants with hepatitis B (positive hepatitis B surface antigen) or hepatitis C infection (positive hepatitis C antibody or HCV RNA); (3) pregnant women; (4) use of steatogenic medication for more than 90 days (such as amiodarone, corticosteroids, methotrexate, valproate and tamoxifen). The patient selection process is illustrated in Fig. 1.

### Data collection

Demographic, physical examination, laboratory blood test, and medical history data of participants were collected. (1) Demographic data included age, gender, race, education level, smoking status, drinking status and income -poverty ratio (PIR). Race was divided into five sections of Mexican America, non-Hispanic white, non-Hispanic black, other Hispainc and other. Educational levels were classified as less than high school, high school or equivalent, and college or above. PIR are divided into 0–1.3, 1.31–3.5, or > 3.5. Smoking status was defined as never smoker, former smoker and current smoker. Drinking status was recorded as non-drinkers, mild to moderate drinkers. (2) Physical examination included BMI, systolic blood pressure (SBP), weight, height, and WC. BMI was calculated as weight in kilograms divided by height in meters squared. SBP is calculated as the average of four measurements. (3) Clinical indicators such as aspartate aminotransferase (ALT), alanine aminotransferase (AST), gamma-glutamyltransferase (GGT), TG, total cholesterol (TC), FBG and glycohemoglobin (HbA1c) were collected. (4) Medical history included diabetes, hypertension and family history of heart disease. The definition of diabetes was self-reported diagnosis, use of insulin or oral hypoglycemic agents, fasting glucose ≥ 7 mmol/L, or HbA1c ≥ 6.5%. The definition of hypertension was self-reported diagnosis or based on multiple measurements, the average SBP ≥ 140 mmHg, and the average diastolic blood pressure ≥ 90 mmHg. Family history of heart disease was established by self-reported physician diagnoses using a standardized medical condition questionnaire. The participants were asked: “Close relative had heart attack?” or “Blood relative w/hypertension/stroke?” or “Blood relatives have angina” and answered by yes or no.

### Assessment of TyG index and TyG‑WHtR

The TyG index was calculated as Ln [fasting TG (mg/dL) × fasting glucose (mg/dL)/2]. TyG-WHtR = TyG × WC/ height. The participants were classifed into four groups (Q1, Q2, Q3, Q4) by the quartiles of TyG index or TyG-WHtR, and the Q1 group was used as the reference group.

### Outcome definitions

We linked the National Death Index (NDI) from the National Center for Health Statistics (NCHS) to obtain the survival status of the participants. Moreover, we utilized the International Statistical Classification of Diseases, 10th Revision (ICD-10) to identify disease-specific deaths, with the NCHS categorizing heart diseases (ICD-10 codes 054–064), malignant neoplasms (ICD-10 codes 019–043), and all other causes (ICD-10 code 010) for our study.

The diagnosis of CVD was established by self-reported physician diagnoses using a standardized medical condition questionnaire. The participants were asked, “Has a doctor or other health professional ever told [you/SP] that [you/s/he]…had congestive heart failure/coronary heart disease/angina pectoris?” A person was considered to have CVD if they answered “yes” to any of the aforementioned questions.

### Statistical analysis

Due to the intricate sampling design of NHANES, our analyses incorporated sample weights, clustering, and stratification to fulfill the necessary criteria for analyzing NHANES data. Study population characteristics were stratified into four groups based on quartiles (Q1-Q4) of the TyG index or TyG-WHtR. Baseline characteristics were presented as median (25th-75th percentile) for continuous variables and as number (percentage) for categorical variables. The four groups were compared using analysis of variance (ANOVA) or the Kruskal-Wallis test for continuous variables, and the χ2 test for categorical variables.

Three models were established with incremental degrees of adjustment for potential confounders of outcome: crude was unadjusted, model 1 was adjusted for age, gender and race, model 2 was adjusted for age, gender, race, smoke, education, PIR, SBP, TC, family history of heart disease and diabetes. To evaluate the independent predictive value of the TyG index and TyG-WHtR, we established multivariable Cox proportional hazards regression models and binary weighted logistic regression models. The Cox proportional hazards model was used to estimate hazard ratios (HRs) and 95% CIs for the association between TyG index and TyG-WHtR and all-cause mortality and CVD mortality. Logistic regression models were used to estimate odds ratios (OR) and 95% confidence intervals (CI) for the association between TyG index and TyG-WHtR and total-CVD, CHF, CHD and angina pectoris. Furthermore, linear trends between TyG and TyG-WHtR quartiles were evaluated using the median value within each quartile as a continuous variable.

To determine whether there was a nonlinear dose-response relationship of the TyG index and TyG-WHtR with the risk of mortality and CVD after multivariable-adjustment, restricted cubic splines (RCS)were fitted, with four knots placed at the 5th, 35th, 65th, and 95th percentiles and the 1% highest and lowest TyG index and TyG-WHtR observations were trimmed. Mediation analyses were used to investigate whether the relevance of TyG and TyG-WHtR to all-cause/ CVD mortality or CVD could be explained by HbA1c, insulin or hypertension after adjusting for covariates in the primary analysis model 2. Stratified analysis was carried out for significant covariates, considering potential effect modifiers such as age, gender, race, PIR, smoke, drink, education, SBP, HbA1c and diabetes. In addition, to evaluate the incremental predictive performance of outcomes following the addition of the TyG index and TyG-WHtR to the basic model, we used the C statistic, continuous net reclassification improvement (NRI), and integrated discrimination improvement (IDI).The statistical analysis was performed using R software (version 4.3.2), and statistical significance was determined using a two-tailed P value of 0.05.

## Results

### Demographic and clinical characteristics in NAFLD and NAFLD with all-cause mortality/CVD mortality/CVD groups

Demographic and clinical characteristics of NAFLD patients and NAFLD with all-cause mortality/CVD mortality/CVD patients were compared in Table 1 and Table S1. Compared with NAFLD patients, NAFLD with all-cause mortality/ CVD mortality/CVD patients tended to be older, male, non-Hispanic white, middle-PIR, smoker, non-drinker and had a family history of CVD. Meanwhile, these groups showed higher levels of SBP, FBG, HbA1c, TyG index and TyG-WHtR and lower levels of BMI, ALT/AST and TC. Interestingly, NAFLD patients with CVD were more likely to be observed in those with a history of diabetes. Conversely, the incidence of all-cause mortality and CVD mortality events in NAFLD patients tended to be higher among those without a history of diabetes.

**Table 1:** Characteristics of NAFLD patients by the presence of all-cause mortality and CVD mortality

	All-cause mortality			CVD mortality		
	**NAFLD** *N* = 5283	**All-cause mortality + NAFLD** *N* = 1335	**P Value** ^2^	**NAFLD** *N* = 5274	**CVD mortality+ NAFLD** *N* = 459	**P Value** ^1^
Age, year	53(40,64)	72(64,79)	< 0.001	53(40,64)	73(64,80)	< 0.001
**Gender, n (%)**			< 0.001			0.001
Female	2,580(49)	544(41)		2,576(49)	188(41)	
Male	2,703(51)	791(59)		2,698(51)	271(59)	
**Race, n (%)**			< 0.001			< 0.001
Mexican America	1,029(19)	206(15)		1,026(19)	69(15)	
Other Hispainc	507(9.6)	62(4.6)		507(9.6)	23(5.0)	
Non-Hispanic white	2,097(40)	784(59)		2,094(40)	263(57)	
Non-Hispanic black	1,231(23)	242(18)		1,230(23)	90(20)	
Other	419(7.9)	41(3.1)		417(7.9)	14(3.1)	
**Education, n (%)**			< 0.001			< 0.001
Less than high school	1,498(28)	563(42)		1,496(28)	187(41)	
High school or equivalent	1,204(23)	323(24)		1,204(23)	109(24)	
College or above	2,579(49)	446(33)		2,572(49)	163(36)	
**Family poverty income ratio, n (%)**			< 0.001			< 0.001
≤ 1.3	1,406(30)	421(35)		1,403(30)	135(32)	
1.31–3.5	1,816(38)	535(44)		1,814(38)	199(47)	
> 3.5	1,541(32)	247(21)		1,537(32)	88(21)	
**Smoke, n (%)**			< 0.001			0.003
Never	95(4.2)	30(3.7)		94(4.2)	13(5.0)	
Former	730(33)	197(25)		729(33)	58(22)	
Now	1,416(63)	574(72)		1,416(63)	189(73)	
**Drink, n (%)**			< 0.001			< 0.001
Never	2,549(48)	790(59)		2,548(48)	263(57)	
Mild-Moderate	2,734(52)	545(41)		2,726(52)	196(43)	
BMI, kg/m^2^	33(29,38)	31(27,35)	< 0.001	33(29,38)	31(27,35)	< 0.001
Systolic blood pressure, mmHg	125(116,137)	133(121,148)	< 0.001	125(116,137)	135(122,153)	< 0.001
ALT/AST	1.07(0.89,1.30)	0.92(0.77,1.10)	< 0.001	1.07(0.89,1.30)	0.92(0.78,1.11)	< 0.001
GGT, U/L	28(20,42)	28(20,45)	0.12	28(20,42)	28(20,46)	0.3
TG, mg/dl	140(99,199)	140(98,205)	0.6	140(99,199)	143(100,213)	0.054
Total cholesterol, mg/dl	193(167,222)	188(161,220)	< 0.001	193(167,222)	187(162,224)	0.12
Fasting Glucose, mg/dl	102(94,118)	111(98,142)	< 0.001	102(94,118)	113(98,150)	< 0.001
Glycohemoglobin, %	5.70(5.40,6.30)	6.00(5.60,6.80)	< 0.001	5.70(5.40,6.30)	6.00(5.60,7.00)	< 0.001
Diabetes, n (%)	1,694(32)	647(48)	< 0.001	1,688(32)	228(50)	< 0.001
Family history of heart disease, n (%)	991(20)	281(22)	0.038	990(20)	101(23)	0.072
TyG	8.98(8.59,9.39)	9.05(8.65,9.52)	< 0.001	8.98(8.59,9.39)	9.11(8.71,9.61)	< 0.001
TyG-WHtR	5.93(5.41,6.55)	5.97(5.47,6.57)	0.2	5.93(5.41,6.55)	6.06(5.57,6.62)	0.004

Median(IQR) for continuous; n() for categorical

^1^Wilcoxon rank sum test; Pearson’s Chi-squared test

### Characteristics of NAFLD patients based on the quartile of TyG index and TyG-WHtR

To characterize the baseline traits of NAFLD patients across different TyG and TyG-WHtR levels, we categorized them into four quartiles(Q) according to the TyG and TyG-WHtR values (TyG index: Q1 ≤ 8.60, Q2:8.60-9.00, Q3:9.00-9.42, Q4 > 9.42; TyG-WHtR: Q1 ≤ 5.42, Q2:5.42–5.94, Q3: 5.94–6.55, Q4 > 6.55). The mean TyG index and TyG-WHtR in the enrolled patients was 9.05 ± 0.71 and 6.03 ± 0.90. The characteristics of the NAFLD patients according to quartiles of TyG and TyG-WHtR are shown in Table 2 and Table S2.

**Table 2:** Baseline characteristics according to TyG index quartiles

	TyG				**P Value** ^1^
	**Q1(≤ 8.60)**	**Q2(8.60-9.00)**	**Q3(9.00-9.42)**	**Q4(>9.42)**	
Age, year	54(37,67)	57(42,69)	60(46,70)	59(46,68)	< 0.001
**Gender, n (%)**					0.004
Female	820(49)	792(47)	799(48)	715(44)	
Male	837(51)	877(53)	853(52)	928(56)	
**Race, n (%)**					< 0.001
Mexican America	210(13)	308(18)	324(20)	393(24)	
Other Hispainc	113(6.8)	143(8.6)	160(9.7)	152(9.3)	
Non-Hispanic white	555(33)	744(45)	803(49)	778(47)	
Non-Hispanic black	691(42)	348(21)	249(15)	186(11)	
Other	88(5.3)	126(7.5)	116(7.0)	134(8.2)	
**Education, n (%)**					< 0.001
Less than high school	442(27)	482(29)	554(34)	581(35)	
High school or equivalent	390(24)	373(22)	389(24)	376(23)	
College or above	825(50)	812(49)	706(43)	686(42)	
**Family poverty income ratio, n (%)**					0.003
≤ 1.3	430(28)	446(30)	441(30)	508(34)	
1.31–3.5	609(40)	578(38)	592(40)	574(39)	
> 3.5	476(31)	486(32)	430(29)	395(27)	
**Smoke, n (%)**					0.6
Never	27(4.0)	34(4.5)	34(4.5)	30(3.5)	
Former	215(32)	222(29)	218(29)	273(32)	
Now	428(64)	503(66)	510(67)	549(64)	
**Drink, n (%)**					0.009
Never	792(48)	822(49)	850(51)	876(53)	
Mild-Moderate	865(52)	847(51)	802(49)	767(47)	
BMI, kg/m^2^	33(29,38)	33(29,37)	32(29,37)	32(29,36)	< 0.001
Systolic blood pressure, mmHg	125(115,137)	125(116,137)	127(117,140)	129(120,142)	< 0.001
ALT/AST	1.00(0.83,1.19)	1.04(0.85,1.24)	1.04(0.86,1.27)	1.09(0.90,1.32)	< 0.001
GGT, U/L	25(19,38)	26(19,38)	28(20,42)	32(23,52)	< 0.001
TG, mg/dl	79(66,93)	124(109,139)	172(149,196)	264(210,344)	< 0.001
Total cholesterol, mg/dl	180(154,206)	189(165,217)	196(168,224)	207(179,239)	< 0.001
Fasting Glucose, mg/dl	97(90,105)	101(94,112)	105(96,123)	129(103,192)	< 0.001
Glycohemoglobin, %	5.60(5.30,5.90)	5.70(5.40,6.10)	5.80(5.50,6.30)	6.40(5.70,8.10)	< 0.001
Diabetes, n (%)	289(17)	427(26)	621(38)	1,003(61)	< 0.001
Family history of heart disease, n (%)	1,254(81)	1,252(79)	1,249(80)	1,220(79)	0.5
All-cause mortality, n (%)	295(18)	322(19)	329(20)	387(24)	< 0.001
CVD mortality, n (%)	87(6.0)	103(7.1)	122(8.5)	147(11)	< 0.001
Total-CVD, n (%)	176(11)	224(14)	252(16)	301(19)	< 0.001
CHF, n (%)	75(4.7)	80(4.9)	98(6.1)	126(7.8)	< 0.001
CHD, n (%)	88(5.5)	102(6.3)	116(7.2)	154(9.5)	< 0.001
Angina pectoris, n (%)	48(3.0)	81(5.0)	90(5.5)	114(7.0)	< 0.001

Median(IQR) for continuous; n() for categorical

CVD mortality, Cardiovascular mortality; Total-CVD, Total cardiovascular disease; CHF, Congestive heart failure; CHD, Coronary heart disease

^1^Kruskal-Wallis rank sum test; Pearson’s Chi-squared test

Patients with higher TyG index are likely to be older, male, non-Hispanic white, middle-PIR, non-drinker, and to be higher SBP, GGT, TG, TC, FBG, HbA1c and lower BMI. Meanwhile, they also exhibited higher rates of all-cause mortality, CVD mortality, diabetes, total-CVD, CHF, CHD and angina pectoris events. Participants with higher TyG-WHtR tended to be female, non-Hispanic white, middle-PIR, smoker, and to have higher BMI, SBP, TG, TC, FBG, HbA1c and lower GGT. And higher TyG-WHtR groups demonstrated elevated rates of CVD mortality, diabetes, total-CVD, CHF, CHD and angina pectoris events.

#### Associations of TyG index and TyG-WHtR with all-cause mortality/CVD mortality/total-CVD/CHF/CHD/angina pectoris

**Fig. 2:**
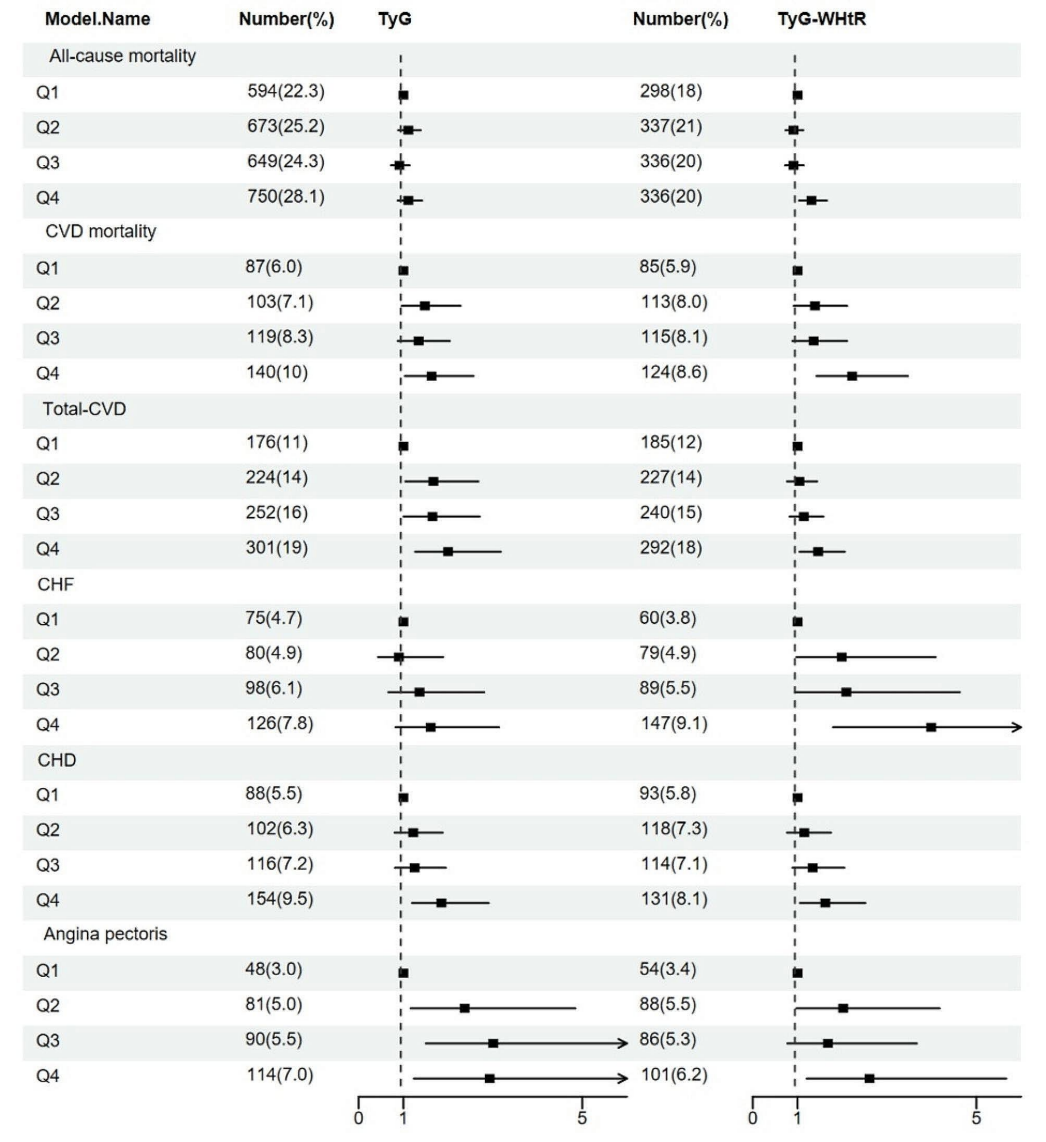
Forest plot of the TyG index and TyG-WHtR association with all-cause mortality, CVD mortality, total-CVD, congestive heart failure, coronary heart disease and angina pectoris

Figure 2 illustrates the relationship of TyG and TyG-WHtR with mortality and cardiovascular disease (CVD). Detailed information on all the aforementioned associations is available in Table S3-S8. After adjusting for age, gender, race, smoke, education, PIR, SBP, TC, family history of heart disease and diabetes in Model 2, the results showed that except for no significant association between TyG and all-cause mortality and CHF, both TyG and TyG-WHtR exhibited significant positive correlations or trends of positive correlation with all-cause mortality, CVD mortality, total-CVD, CHF, CHD and angina pectoris incidence. (p trend < 0.05, TyG-CVD mortality: p trend = 0.07, TyG- angina pectoris: p trend = 0.078)

For all-cause mortality, CVD mortality and CHF, TyG-WHtR had higher predictive power (HR:1.31, 95%CI 1.03–1.66; HR:2.22, 95%CI 1.42–3.47; OR:3.99, 95%CI 1.79–8.93). And TyG index demonstrated a stronger association with total-CVD, CHD and angina pectoris (OR:2.00, 95%CI 1.26–3.18; OR:1.85, 95%CI 1.19–2.91; OR:2.93, 95%CI 1.23-7.00).

#### Using RCS analysis to explore the relationship between TyG and TyG-WHtR, and all-cause mortality/CVD mortality/Total-CVD/CHF/CHD and angina pectoris

**Fig. 3:**
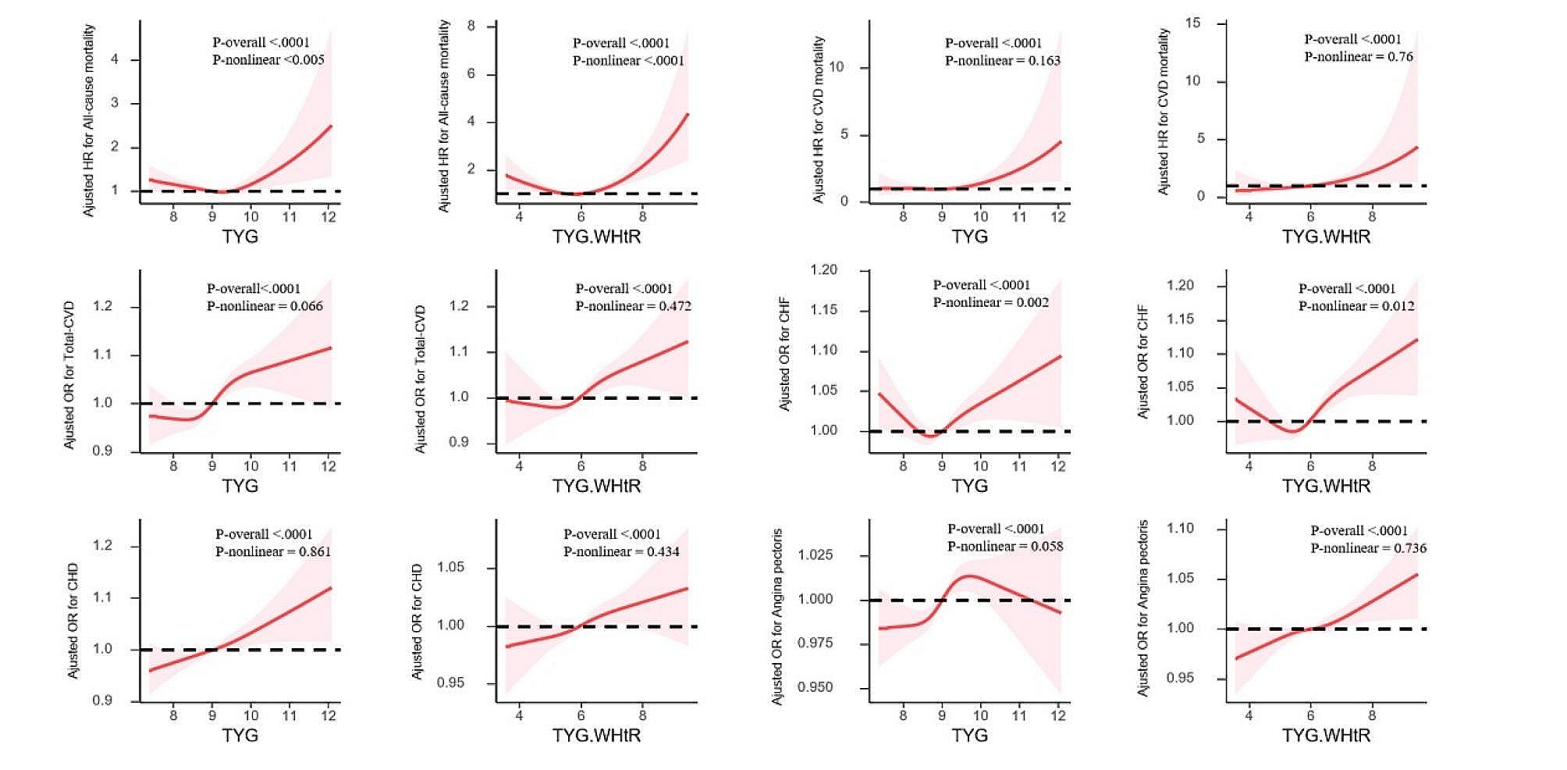
Associations between TyG and TyG-WHtR with all-cause mortality, CVD mortality, total-CVD, congestive heart failure, coronary heart disease and angina pectoris were evaluated by RCS after adjustment for the covariables

Figure 3 shows the association between TyG, TyG-WHtR, and all-cause mortality, CVD mortality, total-CVD, CHF, CHD and angina pectoris using RCS analysis. After adjusting for all covariates in the master analytical model 2 above, a linear correlation was observed between TyG, TyG-WHtR and CVD mortality, total-CVD, CHD and angina pectoris (P-overall < 0.0001, P-nonlinear > 0.05). In contrast, TyG and TyG-WHtR showed nonlinear associations with all-cause mortality and CHF (P-overall < 0.0001, P-nonlinear < 0.05). Therefore, it is evident that both excessively high and low levels of TyG and TyG-WHtR increase the risk of all-cause mortality and the incidence of CHF in NAFLD patients.

#### Mediation analysis of TyG, TyG-WHtR and all-cause mortality, CVD mortality, total-CVD, CHF, CHD and angina pectoris

Mediation analyses indicated that HbA1c, insulin, and hypertension indirectly mediated the associations between TyG, TyG-WHtR and all-cause mortality, CVD mortality, total CVD, CHF, CHD and angina pectoris (Fig. S1). For TyG, the HbA1c-mediated indirect effects of CVD mortality, total-CVD, and CHD accounted for 40.9, 25.5, and 26.1%, respectively; for TyG-WHtR, the HbA1c-mediated effects of all-cause mortality, total-CVD, CHF, CHD and angina pectoris were − 76.7, 26.3, 9.9, 59.8, and 32.5%, respectively. The indirect effects of the insulin-mediated association of TyG with all-cause mortality, CVD mortality, total-CVD, CHF, and CHD accounted for 9.9, 2.9, 0.4, 5.6 and 2.6%, respectively. The indirect effects of the insulin-mediated association of TyG-WHtR with all-cause mortality, total-CVD, CHF, and CHD accounted for − 18.9, 7.5, 5.6, and 11.7%, respectively.

For TyG, the hypertension-mediated indirect effects of all-cause mortality, CVD mortality, total-CVD, CHF, CHD and angina pectoris accounted for 7.8, 0.8, 7.8, 9.1, 4.9 and 7.7%, respectively. The percentages of indirect effects for the association between hypertension-mediated TyG-WHtR and all-cause mortality, total-CVD, CHF, CHD, and angina pectoris were − 15.7, 7.3, 15.4, 23.3 and 20.1%, respectively.

#### Stratification of TyG, TyG‑WHtR in relation to all-cause mortality, CVD mortality, total-CVD, CHF, CHD and angina pectoris. angina pectoris, and coronary heart disease

After controlling for variables, stratified analyses based on age, gender, PIR, race, smoking, drinking, education, SBP, HbA1c, and diabetes (Table S9-S20)indicated that significant associations of TyG with total-CVD events, and TyG-WHtR with CVD mortality, and CHF were more frequently observed in individuals aged < 60 years; Significant correlations of TyG-WHtR with total-CVD events, CHF, and angina pectoris were more likely to be observed in individuals with HbA1c ≥ 6.5; In addition, the significance of TyG with total-CVD events was more frequent in individuals without diabetes, whereas the significance of TyG-WHtR with angina pectoris was more frequent in individuals with diabetes.

### Incremental predictive value of TyG index and TyG-WHtR

**Fig. 4:**
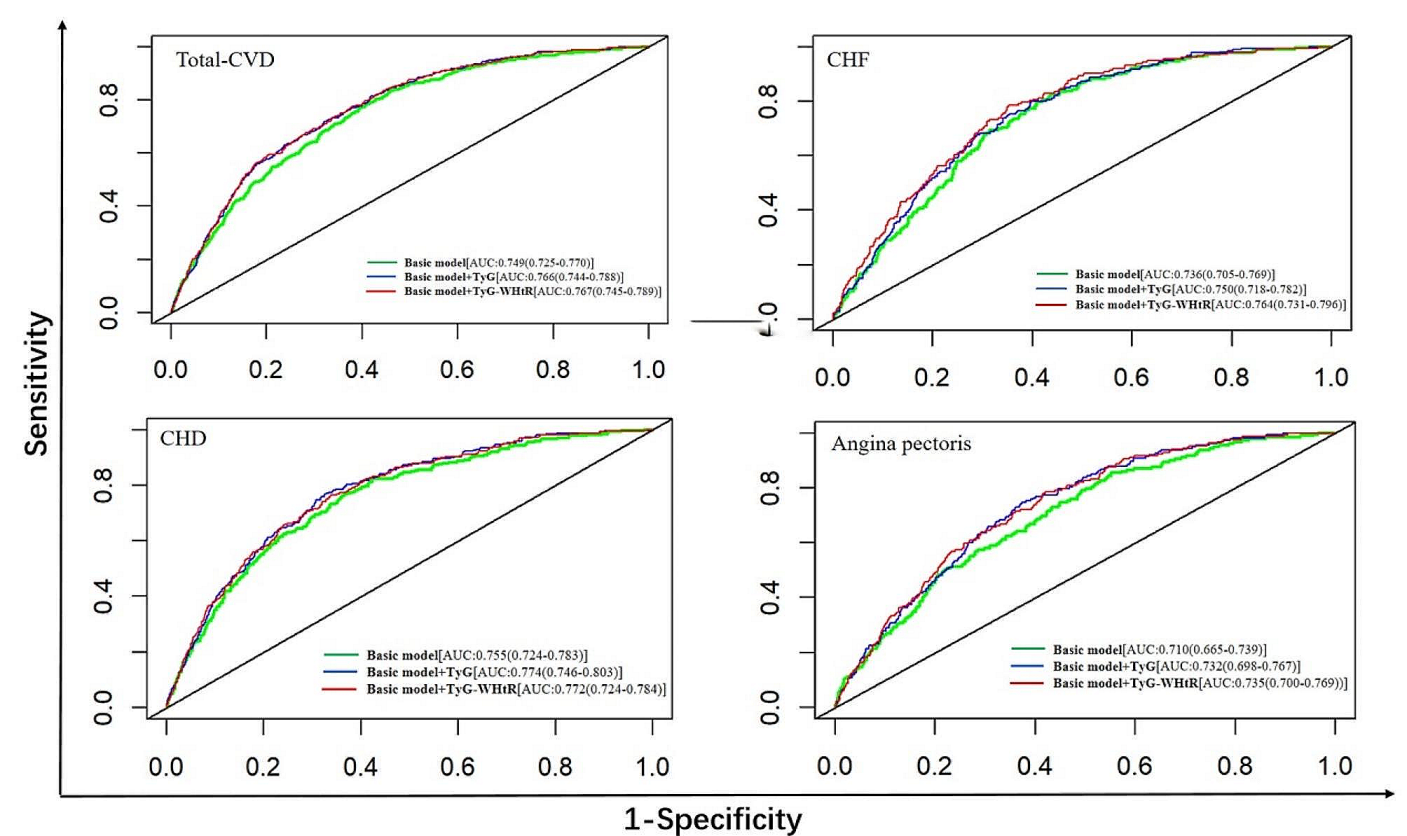
ROC Curve analysis for TyG index and TyG-WHtR predicted total-CVD, congestive heart failure, coronary heart disease and angina pectoris

The incremental predictive values of the TyG index and TyG-WHtR for all-cause mortality, CVD mortality, and the mentioned CVDs are summarized in Table 3. The addition of the TyG index and TyG-WHtR significantly improved the C-statistics of the base model (except for the added TyG index for CHF, all others *p* < 0.05). Moreover, the improvement capabilities of the TyG and TyG-WHtR are comparable **(**Fig. 4; Table 3**).** Additionally, risk reclassification and discriminative power also improved for most outcomes after the inclusion of these two indices in the base model.

**Table 3:** Incremental predictive value of the cumulative TyG index

	C-statistic (95%CI)	*P* value	Continunous NRI (95%CI)	*P* value	IDI(95%CI)	*P* value
**All-cause mortality**						
Basic model	0.810(0.798–0.821)		ref		ref	
Basic model + TyG	0.812(0.800-0.823)	0.004	0.267(-0.011-0.462)	0.054	0.020(-0.024-0.046)	0.07
Basic model + TyG-WHtR	0.810(0.790–0.825)	0.01	0.185(-0.093-0.40	0.08	0.005(-0.001-0.014)	0.08
**CVD mortality**						
Basic model	0.812(0.800-0.824)		ref		ref	
Basic model + TyG	0.813(0.802–0.825)	0.01	0.142(-0.277-0.375)	0.18	0.003(-0.006-0.015)	0.26
Basic model + TyG-WHtR	0.814(0.802–0.826)	0.02	0.127(0.022–0.195)	0.01	0.005(0.001–0.011)	0.01
**Total-CVD**						
Basic model	0.749(0.725–0.770)		ref		ref	
Basic model + TyG	0.766(0.744–0.788)	< 0.001	0.162(0.094–0.230)	< 0.001	0.002(0.0006–0.004)	0.008
Basic model + TyG-WHtR	0.767(0.745–0.789)	< 0.001	0.190(0.122–0.259)	< 0.001	0.004(0.001–0.006)	0.001
**CHF**						
Basic model	0.736(0.705–0.769)		ref		ref	
Basic model + TyG	0.750(0.718–0.782)	0.08	0.173(0.071–0.276)	< 0.001	0.007(-0.005-0.002)	0.23
Basic model + TyG-WHtR	0.764(0.731–0.796)	0.01	0.311(0.208–0.414)	< 0.001	0.008((0.003–0.012)	< 0.001
**CHD**						
Basic model	0.755(0.724–0.784)		ref		ref	
Basic model + TyG	0.775(0.746–0.803)	0.003	0.235(0.142–0.329)	< 0.001	0.005(0.002–0.007)	0.003
Basic model + TyG-WHtR	0.772(0.724–0.784)	0.005	0.170(0.077–0.265)	< 0.001	0.002(0.0004–0.004)	0.04
**Angina pectoris**						
Basic model	0.710(0.665–0.739)		ref		ref	
Basic model + TyG	0.733(0.698–0.767)	0.019	0.149(0.040–0.259)	0.007	0.002(0.0001–0.003)	0.04
Basic model + TyG-WHtR	0.735(0.700-0.769)	0.02	0.129(0.020–0.239)	0.02	0.002(0.0003–0.004)	0.02

The conventional model was adjusted for age, gender, race, smoke, education, income-poverty ratio, systolic blood pressure, total cholesterol, family history of heart disease and diabetes

CI, confidence interval; IDI, integrated discrimination improvement; NRI, net reclassification improve; CVD mortality, cardiovascular mortality; CHF, chronic heart failure; CHD, coronary heart disease

## Discussion

Our analysis showed that except for no significant association between TyG and CVD mortality and CHF, both TyG and TyG-WHtR were positively associated with all-cause mortality, CVD mortality, total-CVD, CHF, CHD and angina pectoris (*P* < 0.05). In particular, TyG showed a better correlation with total-CVD, CHD, and angina, whereas TyG-WHtR showed a higher association with all-cause mortality, CVD mortality and CHF.

The TyG index has a high sensitivity and specificity in detecting IR [8, 21]. The reason for TyG index to effectively predict IR may be that glucotoxicity and lipotoxicity are key mechanisms to mediate IR [21]. After adjusting for covariates, our study showed that TyG and TyG-WHtR were significantly and positively associated with all-cause mortality, CVD mortality, total-CVD, CHF, angina pectoris, and CHD. RCS analysis showed that most of the aforementioned associations were linear after adjustment for all covariates. However, in patients with NAFLD, TyG and TyG-WHtR were nonlinearly correlated with all-cause mortality and CHF, indicating that both excessively high and low levels of TyG and TyG-WHtR increase the risk for these outcomes. The nonlinear relationship between TyG and all-cause mortality is consistent with the findings of the previous studies [22]. This may be related to excessively high or low IR, inflammatory response, oxidative stress and vascular endothelial function [5]. Meanwhile, TyG and TyG-WHtR exhibit non-linear correlations in patients with NAFLD and CHF, which may be attributed to the complexity of the disease, the parameter settings of the RCS curves in statistical analyses, and the choice of statistical model [10, 23–25].

NAFLD is defined as excessive fat accumulation in the liver and elevated levels of free fatty acids are thought to contribute to IR in skeletal muscle [26]. In addition to lipid accumulation, hepatic inflammation, activation of pro-inflammatory cytokines and related transcription factors are key mechanisms that disrupt insulin signaling and contribute to IR [27]. In recent years, it has been established that 90% of NAFLD patients are accompanied by metabolic dysfunction, obesity, and obesity-related diseases such as type 2 diabetes mellitus [28]. Therefore, NAFLD is often characterized by lipotoxicity and metabolic syndromes such as IR, which are predominantly associated with obese individuals [1]. Meanwhile, CVD is a common cause of death in patients with NAFLD, making NAFLD a potential independent predictor of CVD [29, 30]. The related mechanisms of CVD in NAFLD patients have been extensively investigated [2, 31, 32] and mainly involve IR, endothelial dysfunction, glyco-lipotoxicity and oxidative stress. Under physiological conditions, insulin plays an important role in balancing contractile and diastolic functions in vascular endothelial cells through phosphatidylinositol 3 -kinase (PI3K) and mitogen-activated protein kinase (MAPK) dependent signaling pathways. However, this balance is disturbed in IR, resulting in endothelial dysfunction and causing glyco-lipotoxicity in the organism. Meanwhile, excessive fat accumulation in the liver promotes the generation of reactive oxygen species (ROS) in the mitochondria, resulting in large amounts of oxidized low-density lipoproteins in the circulation, leading to endothelial oxidative stress injury and impaired vascular function [33, 34]. These abnormal physiological processes are common in NAFLD patients, which partially explain the high incidence of CVD. Meanwhile, a meta-analysis of 34,043 patients with NAFLD showed an increased risk of lethal and non-lethal CVD in patients with NAFLD compared with non-NAFLD patients [35]. Therefore, it is useful to combine the TyG index and indicators of obesity to predict the occurrence of CVD and death in patients with NAFLD.

In numerous studies, TyG and its combined obesity-related indices, such as TyG-BMI, TyG-WC, TyG-WHR, and TyG-WHtR can be used as surrogate indices for IR, which are associated with increased risk of NAFLD and other metabolic disorders [17, 36, 37], and can better characterize IR status in NAFLD patients [38, 39]. However, an Indian study in 2023 showed that TyG is not superior to FIB-4 for the assessment of fibrosis in NAFLD patients [40]. Therefore, it is prudent to assess the degree of liver fibrosis in patients with NAFLD using the TyG correlation index. In addition, TyG and its combined obesity-related indicators can better reflect cardiovascular events. A cross-sectional study including 424 patients with NAFLD showed that TyG and TyG-BMI were risk factors for CHD in patients with NAFLD after adjustment for age, sex, hypertension, and diabetes mellitus [9]. A study from NHANES 2003–2018 indicated that the correlation and diagnostic efficacy of TyG-WC and TyG-WHtR in relation to CVD and death was superior to TyG index to some extent [5]. Moreover, TyG and its related indices are also associated with NAFLD patients in adolescents. Two Korean retrospective analyses of participants aged 10–19 years showed that TyG and TyG-WHtR were predictors of NAFLD in adolescents and TyG-WHtR was superior to TyG, especially in females [41, 42]. The exact mechanism of the close relationship between the TyG index and NAFLD and CVD has not been fully elucidated. However, factors such as IR, endothelial dysfunction, inflammation, dysregulated glycolipid metabolism and thrombosis are known to be involved [43, 44]. These factors may explain the occurrence of many CVDs in NAFLD patients with elevated TyG levels.

Among these TyG-related indices, BMI cannot distinguish between increases due to muscle and fat. Higher muscle mass may reduce the risk of premature death. Thus, although a higher BMI is associated with an increased risk of morbidity and mortality, this correlation requires further verification. Therefore, we chose TyG-WHtR as an indicator of obesity to predict cardiovascular prognosis in NAFLD patients because of its ease of acquisition, high efficacy, and ability to reflect body fat accumulation.

Our study is the first to investigate the relationship between TyG and the combined obesity indicator TyG-WHtR and all-cause mortality, CVD mortality, total-CVD, CHF, CHD, and angina pectoris in NAFLD patients. Significant associations of TyG with total-CVD events and TyG-WHtR with CVD mortality, and CHF were more frequently observed in individuals aged < 60 years; significant correlations of TyG-WHtR with totalCVD events, CHF, and angina pectoris were more easily observed in individuals with HbA1c ≥ 6.5; In addition, the association of TyG with total CVD events was predominant among individuals without diabetes, whereas the association of TyG-WHtR with angina pectoris was more pronounced among those with diabetes. Some findings also revealed that the association of TyG, TyG-WHtR with CVD and CVD mortality was higher in the absence of diabetes [5], which may be related to the use of hypoglycemic drugs in diabetic patients and directly affect the TyG index by lowering blood glucose levels. Moreover, a study involving 7,521 Iranian individuals found that the TyG index was significantly associated with CVD risk, particularly in younger individuals [25]. Additionally, a study based on NHAENS reported that TyG and TyG-WHtR were more strongly associated with cardiovascular events and CVD mortality in younger patients [5]. Similarly, another study of the US population found that the TyG index was more strongly associated with HF in patients under 60 years old, which aligns with our findings [45]. This may be due to the relatively diminished predictive power of TyG and TyG-WHtR with increasing age, likely because of the accumulation of additional CVD risk factors. There have been conflicting reports on gender differences in TyG, TyG-WHtR and CVD, CHF, CHD and CVD mortality [46, 47]. These discrepancies may be related to socio-economic differences between genders. Overall, men exhibited a slightly higher event rate, which may be related to their greater exposure to chemicals and environmental factors that could amplify their association with CVD [25].

Our study also demonstrated that HbA1c, insulin, and hypertension partially mediate the association between TyG, TyG-WHtR, and CVD mortality, total-CVD, CHF, angina pectoris, and CHD. Our findings revealed that hypertension primarily mediated the association between TyG-WHtR and CHF, while the majority of the remaining associations were largely mediated by HbA1c. This suggests that effective interventions can be developed to target these mediators in order to reduce the risk of CVD in patients with NAFLD.

### Strengths and limitations

The strength of this study lies in the adjustment of covariates to investigate the relationships between TyG, TyG-WHtR, and various outcomes including all-cause mortality, CVD mortality, total-CVD, CHF, angina pectoris, and CHD. Furthermore, the study examines the intermediate roles of HbA1c, insulin, and hypertension in the associations between TyG and TyG-WHtR with these diseases. This provides valuable clinical guidance for the management of patients with NAFLD who also have CVDs. However, there are also some limitations to this study. Firstly, data on CVD were collected from participants by self-reporting, which may have some false positives or the omission of patients with undiagnosed CVD. Secondly, our study is based on the data from the United States, and further research is necessary to investigate whether these discoveries can be widely applied to other regions. Thirdly, although TyG and TyG-WHtR can be used as surrogate indicators of visceral fat, they do not take into account metabolic factors and may have limited application [18].Finally, according to our study, although TyG and TyG-WHtR can be used as preliminary predictors of cardiovascular events and mortality in patients with NAFLD, their diagnostic value needs to be further investigated.

## Conclusions

The study revealed that patients with NAFLD and CVD exhibited elevated levels of both TyG and TyG-WHtR. Additionally, TyG-WHtR can serve as a simple complementary indicator alongside TyG to predict CVD mortality, as well as conditions such as CHF, angina pectoris, and CHD.Furthermore, the two indices can be widely used in primary hospitals and communities for screening and prediction in patients with potential NAFLD combined with CVD due to their simplicity and inexpensiveness.

### Electronic supplementary material

Below is the link to the electronic supplementary material.

Supplementary Material 1

Supplementary Material 2

## Data Availability

The data were obtained from publicly available sources.

## Abbreviations

TyG: Triglyceride-glucose index
CVD: Cardiovascular disease
NAFLD: Non-alcoholic fatty liver disease
NHANES: National Health and Nutrition Examination Survey
RCS: Restricted cubic spline
ROC: Receiver operating characteristic
ANOVA: Analysis of variance
CHF: Chronic heart failure
IR: Insulin resistance
WC: Waist circumference
WHR: Waist-to-hip ratio
WHtR: Waist-to-height ratio
CHD: Coronary Heart Disease
USFLI: US fatty liver index
AUROC: Area under the receiver operating characteristic curve
PIR: Income -poverty ratio
SBP: Systolic blood pressure
ALT: Aspartate aminotransferase
AST: Alanine aminotransferase
GGT: Gamma-glutamyltransferase
TG: Triglycerides
TC: Total cholesterol
FBG: Fasting blood glucose
HbA1c: Glycohemoglobin
NDI: National Death Index
NCHS: National Center for Health Statistics
ICD-10: International Statistical Classification of Diseases,10th Revision
HR: Hazard ratio
OR: Odds ratios
CI: Confidence intervals
NRI: Net reclassification improvement
IDI: Integrated discrimination improvement
PI3K: Phosphatidylinositol 3 -kinase
MAPK: Mitogen-activated protein kinase
ROS: Reactive oxygen species

## References

[1] Grander C, Grabherr F, Tilg H. Non-alcoholic fatty liver disease: pathophysiological concepts and treatment options. [Cardiovasc Res. 2023;119(9):1787–98. 37364164 10.1093/cvr/cvad095 PMC10405569

[2] Targher G, Byrne CD, Tilg H. NAFLD and increased risk of cardiovascular disease: clinical associations, pathophysiological mechanisms and pharmacological implications. [Gut. 2020;69(9):1691–705. 32321858 10.1136/gutjnl-2020-320622

[3] Keen H, Jarrett RJ, Alberti KG. Diabetes mellitus: a new look at diagnostic criteria. [Diabetologia. 1980;18(1):81. 7364164 10.1007/BF01228309

[4] Damen JA, Hooft L, Schuit E, et al. Prediction models for cardiovascular disease risk in the general population. Syst review[Bmj. 2016;353:i2416. 10.1136/bmj.i2416 PMC4868251 27184143

[5] Dang K, Wang X, Hu J, et al. The association between triglyceride-glucose index and its combination with obesity indicators and cardiovascular disease: NHANES 2003–2018[. Cardiovasc Diabetol. 2024;23(1):8. 38184598 10.1186/s12933-023-02115-9 PMC10771672

[6] Banerjee D, Biggs ML, Mercer L, et al. Insulin resistance and risk of incident heart failure. Cardiovasc Health Study[Circ Heart Fail. 2013;6(3):364–70. 10.1161/CIRCHEARTFAILURE.112.000022 PMC3888807 23575256

[7] Alizargar J, Bai CH, Hsieh NC, et al. Use of the triglyceride-glucose index (TyG) in cardiovascular disease patients. [Cardiovasc Diabetol. 2020;19(1):8. 31941513 10.1186/s12933-019-0982-2 PMC6963998

[8] Er LK, Wu S, Chou HH, et al. Triglyceride glucose-body Mass Index is a simple and clinically useful surrogate marker for insulin resistance in nondiabetic Individuals[. PLoS ONE. 2016;11(3):e0149731. 26930652 10.1371/journal.pone.0149731 PMC4773118

[9] Zhao J, Fan H, Wang T, et al. TyG index is positively associated with risk of CHD and coronary atherosclerosis severity among NAFLD. patients[Cardiovasc Diabetol. 2022;21(1):123. 10.1186/s12933-022-01548-y PMC9250269 35778734

[10] Tao LC, Xu JN, Wang TT, et al. Triglyceride-glucose index as a marker in cardiovascular diseases: landscape and limitations[. Cardiovasc Diabetol. 2022;21(1):68. 35524263 10.1186/s12933-022-01511-x PMC9078015

[11] Huang R, Wang Z, Chen J, et al. Prognostic value of triglyceride glucose (TyG) index in patients with acute decompensated heart failure. [Cardiovasc Diabetol. 2022;21(1):88. 35641978 10.1186/s12933-022-01507-7 PMC9158138

[12] Wormser D, Kaptoge S, Di Angelantonio E, et al. Separate and combined associations of body-mass index and abdominal adiposity with cardiovascular disease: collaborative analysis of 58 prospective studies. [Lancet. 2011;377(9771):1085–95. 21397319 10.1016/S0140-6736(11)60105-0 PMC3145074

[13] Yusuf S, Anand S. Body-mass index, abdominal adiposity, and cardiovascular. risk[Lancet. 2011;378(9787):226–7. author reply 228. 10.1016/S0140-6736(11)61120-3 21763930

[14] Tian X, Chen S, Wang P, et al. Insulin resistance mediates obesity-related risk of cardiovascular disease: a prospective cohort study. [Cardiovasc Diabetol. 2022;21(1):289. 36564775 10.1186/s12933-022-01729-9 PMC9789633

[15] Jayedi A, Soltani S, Zargar MS, et al. Central fatness and risk of all cause mortality: systematic review and dose-response meta-analysis of 72 prospective cohort studies. [Bmj. 2020;370:m3324. 32967840 10.1136/bmj.m3324 PMC7509947

[16] Sheng G, Lu S, Xie Q, et al. The usefulness of obesity and lipid-related indices to predict the presence of non-alcoholic fatty liver disease[. Lipids Health Dis. 2021;20(1):134. 34629059 10.1186/s12944-021-01561-2 PMC8502416

[17] Malek M, Khamseh ME, Chehrehgosha H, et al. Triglyceride glucose-waist to height ratio: a novel and effective marker for identifying hepatic steatosis in individuals with type 2 diabetes mellitus. [Endocrine. 2021;74(3):538–45. 34355342 10.1007/s12020-021-02815-w

[18] Li X, Sun M, Yang Y, et al. Predictive effect of triglyceride glucose-related parameters, obesity indices, and lipid ratios for diabetes in a Chinese Population: a prospective cohort Study[. Front Endocrinol (Lausanne). 2022;13:862919. 35432185 10.3389/fendo.2022.862919 PMC9007200

[19] Xuan W, Liu D, Zhong J, et al. Impacts of triglyceride glucose-Waist to height ratio on diabetes incidence: a secondary analysis of a Population-based Longitudinal Data[. Front Endocrinol (Lausanne). 2022;13:949831. 35937805 10.3389/fendo.2022.949831 PMC9354460

[20] Ruhl CE, Everhart JE. Fatty liver indices in the multiethnic United States National Health and Nutrition Examination Survey[. Aliment Pharmacol Ther. 2015;41(1):65–76. 25376360 10.1111/apt.13012

[21] Du T, Yuan G, Zhang M, et al. Clinical usefulness of lipid ratios, visceral adiposity indicators, and the triglycerides and glucose index as risk markers of insulin resistance[. Cardiovasc Diabetol. 2014;13:146. 25326814 10.1186/s12933-014-0146-3 PMC4209231

[22] Zhang Q, Xiao S, Jiao X, et al. The triglyceride-glucose index is a predictor for cardiovascular and all-cause mortality in CVD patients with diabetes or pre-diabetes: evidence from NHANES 2001–2018[. Cardiovasc Diabetol. 2023;22(1):279. 37848879 10.1186/s12933-023-02030-z PMC10583314

[23] Wang X, Xu W, Song Q, et al. Association between the triglyceride-glucose index and severity of coronary artery disease. [Cardiovasc Diabetol. 2022;21(1):168. 36050734 10.1186/s12933-022-01606-5 PMC9438180

[24] Zhou Q, Yang J, Tang H, et al. High triglyceride-glucose (TyG) index is associated with poor prognosis of heart failure with preserved ejection fraction. [Cardiovasc Diabetol. 2023;22(1):263. 37775762 10.1186/s12933-023-02001-4 PMC10541699

[25] Barzegar N, Tohidi M, Hasheminia M, et al. The impact of triglyceride-glucose index on incident cardiovascular events during 16 years of follow-up: Tehran lipid and glucose study. Cardiovasc Diabetol. 2020;19(1):155. 32993633 10.1186/s12933-020-01121-5 PMC7526412

[26] Randle PJ, Garland PB, Hales CN, et al. The glucose fatty-acid cycle. Its role Insulin Sensit Metabolic Disturbances Diabetes mellitus[Lancet. 1963;1(7285):785–9. 10.1016/s0140-6736(63)91500-9 13990765

[27] Saltiel AR, Olefsky JM. Inflammatory mechanisms linking obesity and metabolic disease[. J Clin Invest. 2017;127(1):1–4. 28045402 10.1172/JCI92035 PMC5199709

[28] Tilg H, Adolph TE, Dudek M, et al. Non-alcoholic fatty liver disease: the interplay between metabolism. Microbes immunity[Nat Metab. 2021;3(12):1596–607. 10.1038/s42255-021-00501-9 34931080

[29] Tana C, Ballestri S, Ricci F et al. Cardiovascular Risk in non-alcoholic fatty liver disease: mechanisms and therapeutic Implications[. Int J Environ Res Public Health 2019;16(17). 10.3390/ijerph16173104 PMC6747357 31455011

[30] Ma J, Hwang SJ, Pedley A, et al. Bi-directional analysis between fatty liver and cardiovascular disease risk factors[. J Hepatol. 2017;66(2):390–7. 27729222 10.1016/j.jhep.2016.09.022 PMC5250546

[31] Kim JA, Montagnani M, Koh KK, et al. Reciprocal relationships between insulin resistance and endothelial dysfunction: molecular and pathophysiological mechanisms. [Circulation. 2006;113(15):1888–904. 16618833 10.1161/CIRCULATIONAHA.105.563213

[32] Potenza MA, Marasciulo FL, Chieppa DM, et al. Insulin resistance in spontaneously hypertensive rats is associated with endothelial dysfunction characterized by imbalance between NO and ET-1 production[. Am J Physiol Heart Circ Physiol. 2005;289(2):H813–822. 15792994 10.1152/ajpheart.00092.2005

[33] Yesilova Z, Yaman H, Oktenli C, et al. Systemic markers of lipid peroxidation and antioxidants in patients with nonalcoholic fatty liver disease[. Am J Gastroenterol. 2005;100(4):850–5. 15784031 10.1111/j.1572-0241.2005.41500.x

[34] Cavalca V, Cighetti G, Bamonti F, et al. Oxidative stress and homocysteine in coronary artery disease. [Clin Chem. 2001;47(5):887–92. 11325893 10.1093/clinchem/47.5.887

[35] Targher G, Byrne CD, Lonardo A, et al. Non-alcoholic fatty liver disease and risk of incident cardiovascular disease: a meta-analysis[. J Hepatol. 2016;65(3):589–600. 27212244 10.1016/j.jhep.2016.05.013

[36] Behboudi-Gandevani S, Ramezani Tehrani F, Cheraghi L, et al. Could a body shape index and waist to height ratio predict insulin resistance and metabolic syndrome in polycystic ovary syndrome?[. Eur J Obstet Gynecol Reprod Biol. 2016;205:110–4. 27579518 10.1016/j.ejogrb.2016.08.011

[37] Hou X, Chen S, Hu G, et al. Stronger associations of waist circumference and waist-to-height ratio with diabetes than BMI in Chinese adults[. Diabetes Res Clin Pract. 2019;147:9–18. 30144478 10.1016/j.diabres.2018.07.029

[38] Xue Y, Xu J, Li M, et al. Potential screening indicators for early diagnosis of NAFLD/MAFLD and liver fibrosis: triglyceride glucose index-related parameters. [Front Endocrinol (Lausanne). 2022;13:951689. 36120429 10.3389/fendo.2022.951689 PMC9478620

[39] Lim J, Kim J, Koo SH, et al. Comparison of triglyceride glucose index, and related parameters to predict insulin resistance in Korean adults: an analysis of the 2007–2010 Korean National Health and Nutrition Examination Survey[. PLoS ONE. 2019;14(3):e0212963. 30845237 10.1371/journal.pone.0212963 PMC6405083

[40] Jeji PS, Mishra D, Shukla M et al. Comparison of Triglyceride-glucose Index (TyG index) and FIB-4 for Assessing Fibrosis in NAFLD[*Journal of Clinical and Experimental Hepatology* 2023;13.

[41] Song K, Park G, Lee HS, et al. Comparison of the triglyceride glucose index and modified triglyceride glucose indices to predict nonalcoholic fatty liver disease in Youths[. J Pediatr. 2022;242:79–e8571. 34808224 10.1016/j.jpeds.2021.11.042

[42] Song K, Lee HW, Choi HS et al. Comparison of the modified TyG indices and other parameters to Predict non-alcoholic fatty liver Disease in Youth[*Biology*. (Basel). 2022;11(5). 10.3390/biology11050685 PMC9138077 35625413

[43] Shahavandi M, Djafari F, Shahinfar H, et al. The association of plant-based dietary patterns with visceral adiposity, lipid accumulation product, and triglyceride-glucose index in Iranian adults[. Complement Ther Med. 2020;53:102531. 33066861 10.1016/j.ctim.2020.102531

[44] Wang A, Tian X, Zuo Y, et al. Association between the triglyceride-glucose index and carotid plaque stability in nondiabetic adults[. Nutr Metab Cardiovasc Dis. 2021;31(10):2921–8. 34353702 10.1016/j.numecd.2021.06.019

[45] Wang H, He S, Wang J, et al. Hyperinsulinemia and plasma glucose level independently associated with all-cause and cardiovascular mortality in Chinese people without diabetes-A post-hoc analysis of the 30-year follow-up of Da Qing diabetes and IGT study[. Diabetes Res Clin Pract. 2023;195:110199. 36481224 10.1016/j.diabres.2022.110199

[46] Li X, Chan JSK, Guan B, et al. Triglyceride-glucose index and the risk of heart failure: evidence from two large cohorts and a mendelian randomization analysis. [Cardiovasc Diabetol. 2022;21(1):229. 36329456 10.1186/s12933-022-01658-7 PMC9635212

[47] Qu H, Long LZ, Chen L, et al. Triglyceride-glucose index and estimated 10-year risk of a first hard cardiovascular event[. Front Cardiovasc Med. 2022;9:994329. 36698933 10.3389/fcvm.2022.994329 PMC9868293

