# Supplementary material for: Association of triglyceride glucose-related parameters with all-cause mortality and cardiovascular disease in NAFLD patients: NHANES 1999–2018
Source: Cardiovasc Diabetol. 2024 Jul 18;23:262. doi: 10.1186/s12933-024-02354-4 (PMC11264797; doi:10.1186/s12933-024-02354-4)
Supplement: Supplementary file 1 — Supplementary Material 1 [file 12933_2024_2354_MOESM1_ESM.docx]

**Supplementary Table 1.** Demographic and clinical characteristics of NAFLD patients by the presence of total-CVD, CHF, CHD and angina pectoris.

|  | **Total-CVD** | | | **CHF** | | | **CHD** | | | **Angina pectoris** | | |
| --- | --- | --- | --- | --- | --- | --- | --- | --- | --- | --- | --- | --- |
|  | **NAFLD**  N=5518 | **Total-CVD**  **+NAFLD**  N=954 | **P Value**^1^ | **NAFLD**  N=6104 | **CHF+NAFLD**  N=380 | **P Value**^1^ | **NAFLD**  N=6012 | **CHD+NAFLD**  N=460 | **P Value**^1^ | **NAFLD**  N=6160 | **Angina pectoris**  **+NAFLD**  N=333 | **P Value**^1^ |
| Age,year | 55(42,67) | 69(60,76) | <0.001 | 57(43,68) | 70(61,76) | <0.001 | 57(43,68) | 70(62,77) | <0.001 | 57(43,68) | 68(60,75) | <0.001 |
| Gender,n (%) |  |  | <0.001 |  |  | 0.10 |  |  | <0.001 |  |  | 0.012 |
| Female | 2,691(49) | 367(38) |  | 2,903(48) | 164(43) |  | 2,929(49) | 132(29) |  | 2,934(48) | 135(41) |  |
| Male | 2,827(51) | 587(62) |  | 3,201(52) | 216(57) |  | 3,083(51) | 328(71) |  | 3,226(52) | 198(59) |  |
| Race,n (%) |  |  | <0.001 |  |  | <0.001 |  |  | <0.001 |  |  | <0.001 |
| Mexican America | 1,056(19) | 121(13) |  | 1,129(18) | 51(13) |  | 1,124(19) | 59(13) |  | 1,147(19) | 43(13) |  |
| Other Hispainc | 491(8.9) | 72(7.5) |  | 532(8.7) | 32(8.4) |  | 534(8.9) | 26(5.7) |  | 541(8.8) | 24(7.2) |  |
| Non-Hispanic white | 2,301(42) | 545(57) |  | 2,644(43) | 205(54) |  | 2,541(42) | 300(65) |  | 2,645(43) | 202(61) |  |
| Non-Hispanic black | 1,262(23) | 171(18) |  | 1,354(22) | 81(21) |  | 1,382(23) | 50(11) |  | 1,385(22) | 50(15) |  |
| Other | 408(7.4) | 45(4.7) |  | 445(7.3) | 11(2.9) |  | 431(7.2) | 25(5.4) |  | 442(7.2) | 14(4.2) |  |
| Education,n (%) |  |  | <0.001 |  |  | <0.001 |  |  | 0.2 |  |  | 0.15 |
| Less than high school | 1,621(29) | 345(36) |  | 1,806(30) | 161(42) |  | 1,815(30) | 154(33) |  | 1,861(30) | 115(35) |  |
| High school or equivalent | 1,265(23) | 217(23) |  | 1,395(23) | 92(24) |  | 1,382(23) | 93(20) |  | 1,409(23) | 79(24) |  |
| College or above | 2,628(48) | 391(41) |  | 2,899(48) | 127(33) |  | 2,811(47) | 213(46) |  | 2,886(47) | 139(42) |  |
| Family poverty  income ratio,n (%) |  |  | <0.001 |  |  | <0.001 |  |  | 0.9 |  |  | 0.12 |
| ≤1.3 | 1,445(29) | 316(37) |  | 1,633(30) | 133(39) |  | 1,637(30) | 125(30) |  | 1,669(30) | 103(34) |  |
| 1.31-3.5 | 1,973(40) | 337(39) |  | 2,162(39) | 152(45) |  | 2,140(39) | 167(40) |  | 2,195(40) | 122(41) |  |
| >3.5 | 1,562(31) | 206(24) |  | 1,714(31) | 55(16) |  | 1,642(30) | 127(30) |  | 1,690(30) | 76(25) |  |
| Smoke,n (%) |  |  | <0.001 |  |  | 0.003 |  |  | <0.001 |  |  | 0.11 |
| Never | 109(4.5) | 14(2.4) |  | 121(4.3) | 3(1.4) |  | 114(4.2) | 10(3.4) |  | 118(4.2) | 5(2.5) |  |
| Former | 781(32) | 140(24) |  | 875(31) | 50(23) |  | 864(32) | 56(19) |  | 874(31) | 51(26) |  |
| Now | 1,548(63) | 432(74) |  | 1,819(65) | 162(75) |  | 1,749(64) | 229(78) |  | 1,839(65) | 144(72) |  |
| Drink,n (%) |  |  | <0.001 |  |  | <0.001 |  |  | 0.6 |  |  | 0.008 |
| Never | 2,679(49) | 533(56) |  | 2,990(49) | 229(60) |  | 2,978(50) | 234(51) |  | 3,036(49) | 189(57) |  |
| Mild-Moderate | 2,839(51) | 421(44) |  | 3,114(51) | 151(40) |  | 3,034(50) | 226(49) |  | 3,124(51) | 144(43) |  |
| BMI,kg/m^2^ | 33(29,37) | 32(28,36) | <0.001 | 32(29,37) | 33(29,38) | 0.6 | 32(29,37) | 31(28,35) | <0.001 | 32(29,37) | 32(29,36) | 0.5 |
| Systolic blood pressure,mmHg | 127(117,139) | 129(117,143) | 0.003 | 127(117,139) | 129(117,144) | 0.2 | 127(117,139) | 128(117,142) | 0.2 | 127(117,139) | 128(116,142) | 0.8 |
| ALT/AST | 1.05(0.88,1.28) | 0.95(0.80,1.14) | <0.001 | 1.04(0.87,1.27) | 0.92(0.76,1.11) | <0.001 | 1.04(0.86,1.27) | 0.95(0.80,1.13) | <0.001 | 1.04(0.86,1.27) | 0.96(0.81,1.13) | <0.001 |
| GGT, U/L | 28(20,43) | 28(20,43) | 0.8 | 28(20,42) | 29(19,45) | 0.3 | 28(20,43) | 28(19,42) | 0.8 | 28(20,43) | 28(21,42) | 0.5 |
| TG, mg/dl | 140(99,200) | 145(103,206) | 0.041 | 140(99,199) | 151(103,214) | 0.031 | 140(99,200) | 146(103,211) | 0.10 | 140(99,200) | 151(106,213) | 0.010 |
| Total cholesterol, mg/dl | 195(169,224) | 175(147,206) | <0.001 | 193(167,223) | 176(149,209) | <0.001 | 194(168,223) | 168(144,200) | <0.001 | 193(167,222) | 179(150,211) | <0.001 |
| Fasting Glucose, mg/dl | 102(94,119) | 113(99,143) | <0.001 | 103(95,121) | 113(98,147) | <0.001 | 103(94,121) | 115(100,148) | <0.001 | 103(95,121) | 115(99,143) | <0.001 |
| Glycohemoglobin, % | 5.70(5.40,6.30) | 6.10(5.60,7.00) | <0.001 | 5.80(5.40,6.30) | 6.20(5.70,7.00) | <0.001 | 5.80(5.40,6.30) | 6.10(5.60,7.10) | <0.001 | 5.80(5.40,6.30) | 6.10(5.60,7.00) | <0.001 |
| Diabetes,n (%) | 1,775(32) | 538(56) | <0.001 | 2,091(34) | 227(60) | <0.001 | 2,047(34) | 259(56) | <0.001 | 2,127(35) | 190(57) | <0.001 |
| Family history of  heart disease, n (%) | 1,014(19) | 255(28) | <0.001 | 1,163(20) | 107(30) | <0.001 | 1,146(20) | 122(28) | <0.001 | 1,172(20) | 96(30) | <0.001 |
| TyG | 8.98(8.59,9.39) | 9.12(8.71,9.53) | <0.001 | 8.99(8.60,9.41) | 9.15(8.70,9.58) | <0.001 | 8.99(8.59,9.40) | 9.14(8.69,9.57) | <0.001 | 8.99(8.59,9.41) | 9.16(8.72,9.57) | <0.001 |
| TyG-WHtR | 5.92(5.41,6.52) | 6.12(5.57,6.72) | <0.001 | 5.93(5.41,6.53) | 6.28(5.66,6.96) | <0.001 | 5.94(5.41,6.54) | 6.01(5.52,6.63) | 0.036 | 5.93(5.41,6.54) | 6.09(5.61,6.69) | <0.001 |
| median(IQR) for continuous;n() for categorical | | | | | | | | | | | | |
| ^1^Wilcoxon rank sum test; Pearson's Chi-squared test | | | | | | | | | | | | |

**Supplementary Table 2**. Baseline characteristics according to TyG-WHtR quartiles.

|  | **TyG-WHtR** | | | |  |
| --- | --- | --- | --- | --- | --- |
|  | **Q1(≤5.42)** | **Q2(5.42-5.94)** | **Q3(5.94-6.55)** | **Q4(＞6.55)** | **P Value**^1^ |
| Age,year | 54(40,67) | 58(44,70) | 60(45,70) | 58(44,68) | <0.001 |
| Gender,n (%) |  |  |  |  | <0.001 |
| Female | 512(31) | 730(44) | 836(51) | 1,028(62) |  |
| Male | 1,138(69) | 914(56) | 806(49) | 622(38) |  |
| Race,n (%) |  |  |  |  | <0.001 |
| Mexican America | 272(16) | 306(19) | 322(20) | 329(20) |  |
| Other Hispainc | 114(6.9) | 151(9.2) | 151(9.2) | 152(9.2) |  |
| Non-Hispanic white | 603(37) | 709(43) | 738(45) | 806(49) |  |
| Non-Hispanic black | 489(30) | 360(22) | 331(20) | 291(18) |  |
| Other | 172(10) | 118(7.2) | 100(6.1) | 72(4.4) |  |
| Education,n (%) |  |  |  |  | <0.001 |
| Less than high school | 434(26) | 498(30) | 550(33) | 565(34) |  |
| High school or equivalent | 385(23) | 373(23) | 366(22) | 398(24) |  |
| College or above | 830(50) | 770(47) | 726(44) | 687(42) |  |
| Family poverty  income ratio,n (%) |  |  |  |  | <0.001 |
| ≤1.3 | 357(24) | 420(28) | 467(32) | 567(38) |  |
| 1.31-3.5 | 534(37) | 616(41) | 605(41) | 585(39) |  |
| >3.5 | 569(39) | 468(31) | 402(27) | 342(23) |  |
| Smoke,n (%) |  |  |  |  | <0.001 |
| Never | 43(6.0) | 31(4.1) | 17(2.2) | 34(4.4) |  |
| Former | 249(35) | 225(30) | 212(27) | 240(31) |  |
| Now | 427(59) | 500(66) | 549(71) | 502(65) |  |
| Drink,n (%) |  |  |  |  | <0.001 |
| Never | 739(45) | 789(48) | 876(53) | 913(55) |  |
| Mild-Moderate | 911(55) | 855(52) | 766(47) | 737(45) |  |
| BMI,kg/m^2^ | 28(26,31) | 31(29,34) | 34(31,37) | 39(35,44) | <0.001 |
| Systolic blood pressure,mmHg | 124(115,136) | 126(117,139) | 127(117,141) | 129(119,141) | <0.001 |
| ALT/AST | 1.05(0.86,1.30) | 1.04(0.86,1.27) | 1.03(0.87,1.26) | 1.03(0.86,1.24) | 0.3 |
| GGT, U/L | 30(21,47) | 28(20,43) | 26(19,40) | 27(19,41) | <0.001 |
| TG, mg/dl | 103(76,143) | 135(97,182) | 154(112,220) | 178(130,257) | <0.001 |
| Total cholesterol, mg/dl | 188(161,217) | 193(168,221) | 194(167,224) | 194(168,224) | <0.001 |
| Fasting Glucose, mg/dl | 99(91,108) | 102(94,116) | 105(96,123) | 114(99,159) | <0.001 |
| Glycohemoglobin, % | 5.60(5.30,6.00) | 5.70(5.40,6.10) | 5.80(5.50,6.40) | 6.10(5.60,7.40) | <0.001 |
| Diabetes,n (%) | 325(20) | 482(29) | 619(38) | 901(55) | <0.001 |
| Family history of  heart disease, n (%) | 1,293(84) | 1,254(80) | 1,222(78) | 1,180(76) | <0.001 |
| All-cause mortality, n (%) | 298(18) | 337(21) | 336(20) | 336(20) | 0.2 |
| CVD mortality, n (%) | 85(5.9) | 113(8.0) | 115(8.1) | 124(8.6) | 0.036 |
| Total-CVD, n (%) | 185(12) | 227(14) | 240(15) | 292(18) | <0.001 |
| CHF, n (%) | 60(3.8) | 79(4.9) | 89(5.5) | 147(9.1) | <0.001 |
| CHD, n (%) | 93(5.8) | 118(7.3) | 114(7.1) | 131(8.1) | 0.082 |
| Angina pectoris, n (%) | 54(3.4) | 88(5.5) | 86(5.3) | 101(6.2) | 0.002 |
| median(IQR) for continuous;n() for categorical | | | | | |
| ^1^Kruskal-Wallis rank sum test; Pearson's Chi-squared test | | | | | |
| CVD mortality,Cardiovascular mortality; Total CVD,Total cardiovascular disease; CHF,Congestive heart failure; CHD,Coronary heart disease | | | | | |

**Supplementary Table 3．**HR (95% CI) for All-cause mortality across groups of TyG index and TyG-WHtR.

| **All-cause Mortality** | | | | | | | | | | | | |
| --- | --- | --- | --- | --- | --- | --- | --- | --- | --- | --- | --- | --- |
|  | crude | | | | model1 | | | | model2 | | | |
|  | **HR**^1^ | **95% CI**^1^ | **p-value** | **p trend**^2^ | **HR**^1^ | **95% CI**^1^ | **p-value** | **p trend**^2^ | **HR**^1^ | **95% CI**^1^ | **p-value** | **p trend**^2^ |
| TyG |  |  |  | **<0.001** |  |  |  | 0.078 |  |  |  | 0.99 |
| Q1 | — | — | — |  | — | — | — |  | — | — | — |  |
| Q2 | 1.10 | 0.94, 1.29 | 0.2 |  | 1.02 | 0.87, 1.20 | 0.8 |  | 1.11 | 0.88, 1.39 | 0.4 |  |
| Q3 | 1.16 | 0.99, 1.36 | 0.065 |  | 0.96 | 0.82, 1.13 | 0.6 |  | 0.91 | 0.72, 1.14 | 0.4 |  |
| Q4 | 1.39 | 1.20, 1.62 | <0.001 |  | 1.23 | 1.05, 1.44 | 0.010 |  | 1.11 | 0.87, 1.42 | 0.4 |  |
| TyG-WHtR |  |  |  | **<0.001** |  |  |  | **<0.001** |  |  |  | **0.013** |
| Q1 | — | — | — |  | — | — | — |  | — | — | — |  |
| Q2 | 1.17 | 1.00, 1.36 | 0.054 |  | 0.99 | 0.85, 1.16 | >0.9 |  | 0.91 | 0.73, 1.13 | 0.4 |  |
| Q3 | 1.22 | 1.04, 1.43 | 0.013 |  | 1.11 | 0.95, 1.30 | 0.2 |  | 0.91 | 0.72, 1.14 | 0.4 |  |
| Q4 | 1.35 | 1.15, 1.58 | <0.001 |  | 1.46 | 1.24, 1.72 | <0.001 |  | 1.31 | 1.03, 1.66 | **0.025** |  |
| ^1^HR = Hazard Ratio, CI = Confidence Interval  ^2^Tests for trends based on the variables containing the median values for each quartile.  crude was unadjusted, model 1 was adjusted for age, gender and race, model 2 was adjusted for age, gender, race, smoke, education, PIR, systolic blood pressure, total cholesterol,family history of heart disease, diabetes. | | | | | | | | | | | | |

**Supplementary Table 4**．HR (95% CI) for CVD mortality across groups of TyG index and TyG-WHtR.

| **CVD Mortality** | | | | | | | | | | | | |
| --- | --- | --- | --- | --- | --- | --- | --- | --- | --- | --- | --- | --- |
|  | crude | | | | model1 | | | | model2 | | | |
|  | **HR**^1^ | **95% CI**^1^ | **p-value** | **p trend**^2^ | **HR**^1^ | **95% CI**^1^ | **p-value** | **p trend**^2^ | **HR**^1^ | **95% CI**^1^ | **p-value** | **p trend**^2^ |
| TyG |  |  |  | **<0.001** |  |  |  | **<0.001** |  |  |  | 0.07 |
| Q1 | — | — | — |  | — | — | — |  | — | — | — |  |
| Q2 | 1.21 | 0.91, 1.62 | 0.2 |  | 1.18 | 0.88, 1.57 | 0.3 |  | 1.48 | 0.96, 2.28 | 0.074 |  |
| Q3 | 1.48 | 1.12, 1.95 | 0.005 |  | 1.19 | 0.89, 1.57 | 0.2 |  | 1.34 | 0.87, 2.04 | 0.2 |  |
| Q4 | 1.86 | 1.43, 2.42 | <0.001 |  | 1.62 | 1.23, 2.14 | <0.001 |  | 1.63 | 1.03, 2.57 | **0.036** |  |
| TyG-WHtR |  |  |  | **<0.001** |  |  |  | **<0.001** |  |  |  | **<0.001** |
| Q1 | — | — | — |  | — | — | — |  | — | — | — |  |
| Q2 | 1.41 | 1.06, 1.87 | 0.018 |  | 1.25 | 0.94, 1.66 | 0.13 |  | 1.39 | 0.91, 2.11 | 0.13 |  |
| Q3 | 1.56 | 1.18, 2.06 | 0.002 |  | 1.40 | 1.06, 1.86 | 0.020 |  | 1.36 | 0.88, 2.11 | 0.2 |  |
| Q4 | 1.90 | 1.45, 2.50 | <0.001 |  | 2.17 | 1.63, 2.90 | <0.001 |  | 2.22 | 1.42, 3.47 | **<0.001** |  |
| ^1^HR = Hazard Ratio, CI = Confidence Interval  ^2^Tests for trends based on the variables containing the median values for each quartile.  crude was unadjusted, model 1 was adjusted for age, gender and race, model 2 was adjusted for age, gender, race, smoke, education, PIR, systolic blood pressure, total cholesterol, family history of heart , diabetes. | | | | | | | | | | | | |

**Supplementary Table 5**．OR (95% CI) for Total-CVD across groups of TyG index and TyG-WHtR.

| **Total-CVD** | | | | | | | | | | | | |
| --- | --- | --- | --- | --- | --- | --- | --- | --- | --- | --- | --- | --- |
|  | crude | | | | model1 | | | | model2 | | | |
|  | **OR^1^** | **95% CI^1^** | **p-value** | **p trend^2^** | **OR^1^** | **95% CI^1^** | **p-value** | **p trend^2^** | **OR^1^** | **95% CI^1^** | **p-value** | **p trend^2^** |
| TyG |  |  |  | **<0.001** |  |  |  | **<0.001** |  |  |  | **0.008** |
| Q1 | — | — | — |  | — | — | — |  | — | — | — |  |
| Q2 | 1.27 | 0.92, 1.74 | 0.15 |  | 1.27 | 0.91, 1.79 | 0.2 |  | 1.67 | 1.04, 2.68 | 0.034 |  |
| Q3 | 1.44 | 1.06, 1.94 | 0.019 |  | 1.34 | 0.96, 1.87 | 0.084 |  | 1.65 | 1.00, 2.71 | 0.049 |  |
| Q4 | 1.81 | 1.29, 2.55 | <0.001 |  | 1.88 | 1.31, 2.70 | <0.001 |  | 2.00 | 1.26, 3.18 | 0.003 |  |
| TyG-WHtR |  |  |  | **<0.001** |  |  |  | **<0.001** |  |  |  | **0.005** |
| Q1 | — | — | — |  | — | — | — |  | — | — | — |  |
| Q2 | 1.25 | 0.93, 1.68 | 0.14 |  | 1.17 | 0.86, 1.59 | 0.3 |  | 1.04 | 0.76, 1.44 | 0.8 |  |
| Q3 | 1.22 | 0.89, 1.66 | 0.2 |  | 1.15 | 0.82, 1.62 | 0.4 |  | 1.14 | 0.82, 1.58 | 0.4 |  |
| Q4 | 1.62 | 1.17, 2.26 | 0.004 |  | 2.04 | 1.43, 2.91 | <0.001 |  | 1.46 | 1.04, 2.06 | 0.028 |  |

^1^HR = Hazard Ratio, CI = Confidence Interval

^2^Tests for trends based on the variables containing the median values for each quartile.

crude was unadjusted, model 1 was adjusted for age, gender and race, model 2 was adjusted for age, gender, race, smoke, education, PIR, systolic blood pressure, total cholesterol, family history of heart disease, diabetes.

S**upplementary Table** **6**．OR (95% CI) for congestive heart failure across groups of TyG index and TyG-WHtR.

| **Congestive Heart Failure** | | | | | | | | | | | | |
| --- | --- | --- | --- | --- | --- | --- | --- | --- | --- | --- | --- | --- |
|  | crude | | | | model1 | | | | model2 | | | |
|  | **OR**^1^ | **95% CI**^1^ | **p-value** | **p trend**^2^ | **OR**^1^ | **95% CI**^1^ | **p-value** | **p trend**^2^ | **OR**^1^ | **95% CI**^1^ | **p-value** | **p trend**^2^ |
| TyG |  |  |  | **0.001** |  |  |  | **0.003** |  |  |  | 0.392 |
| Q1 | — | — | — |  | — | — | — |  | — | — | — |  |
| Q2 | 1.07 | 0.67, 1.73 | 0.8 |  | 1.10 | 0.67, 1.80 | 0.7 |  | 0.90 | 0.43, 1.89 | 0.8 |  |
| Q3 | 1.53 | 0.98, 2.38 | 0.064 |  | 1.48 | 0.93, 2.36 | 0.10 |  | 1.36 | 0.66, 2.81 | 0.4 |  |
| Q4 | 1.91 | 1.33, 2.75 | <0.001 |  | 2.04 | 1.39, 3.00 | <0.001 |  | 1.61 | 0.82, 3.14 | 0.2 |  |
| TyG-WHtR |  |  |  | **<0.001** |  |  |  | **<0.001** |  |  |  | **<0.001** |
| Q1 | — | — | — |  | — | — | — |  | — | — | — |  |
| Q2 | 1.60 | 0.97, 2.63 | 0.065 |  | 1.50 | 0.90, 2.50 | 0.12 |  | 1.99 | 0.97, 4.09 | 0.060 |  |
| Q3 | 1.31 | 0.83, 2.05 | 0.2 |  | 1.24 | 0.76, 2.01 | 0.4 |  | 2.09 | 0.94, 4.63 | 0.070 |  |
| Q4 | 2.75 | 1.74, 4.37 | <0.001 |  | 3.30 | 1.99, 5.47 | <0.001 |  | 3.99 | 1.79, 8.93 | <0.001 |  |
| ^1^HR = Hazard Ratio, CI = Confidence Interval  ^2^Tests for trends based on the variables containing the median values for each quartile.  crude was unadjusted, model 1 was adjusted for age, gender and race, model 2 was adjusted for age, gender, race, smoke, education, PIR, systolic blood pressure, total cholesterol,family history of heart, diabetes. | | | | | | | | | | | | |

**Supplementary Table 7**．OR (95% CI) for coronary heart disease across groups of TyG index and TyG-WHtR.

| **Coronary Heart Disease** | | | | | | | | | | | | |
| --- | --- | --- | --- | --- | --- | --- | --- | --- | --- | --- | --- | --- |
|  | crude | | | | model1 | | | | model2 | | | |
|  | **OR**^1^ | **95% CI**^1^ | **p-value** | **p trend**^2^ | **OR**^1^ | **95% CI**^1^ | **p-value** | **p trend**^2^ | **OR**^1^ | **95% CI**^1^ | **p-value** | **p trend**^2^ |
| TyG |  |  |  | **<0.001** |  |  |  | **<0.001** |  |  |  | **0.002** |
| Q1 | — | — |  |  | — | — |  |  | — | — |  |  |
| Q2 | 1.24 | 0.84, 1.83 | 0.3 |  | 1.20 | 0.78, 1.83 | 0.4 |  | 1.22 | 0.80, 1.88 | 0.4 |  |
| Q3 | 1.29 | 0.87, 1.93 | 0.2 |  | 1.13 | 0.73, 1.75 | 0.6 |  | 1.25 | 0.81, 1.95 | 0.3 |  |
| Q4 | 1.95 | 1.26, 3.02 | 0.003 |  | 1.90 | 1.19, 3.02 | 0.007 |  | 1.85 | 1.19, 2.91 | 0.007 |  |
| TyG-WHtR |  |  |  | **0.019** |  |  |  | **<0.001** |  |  |  | **0.005** |
| Q1 | — | — |  |  | — | — |  |  | — | — |  |  |
| Q2 | 1.28 | 0.97, 1.70 | 0.084 |  | 1.18 | 0.88, 1.59 | 0.3 |  | 1.15 | 0.76, 1.75 | 0.5 |  |
| Q3 | 1.23 | 0.93, 1.64 | 0.15 |  | 1.21 | 0.90, 1.63 | 0.2 |  | 1.34 | 0.88, 2.05 | 0.2 |  |
| Q4 | 1.43 | 1.09, 1.89 | 0.011 |  | 1.74 | 1.29, 2.34 | <0.001 |  | 1.62 | 1.05, 2.52 | 0.032 |  |
| ^1^HR = Hazard Ratio, CI = Confidence Interval  ^2^Tests for trends based on the variables containing the median values for each quartile.  crude was unadjusted, model 1 was adjusted for age, gender and race, model 2 was adjusted for age, gender, race, smoke, education, PIR, systolic blood pressure, total cholesterol, family history of heart disease, diabetes. | | | | | | | | | | | | |

**Supplementary Table 8．**OR (95% CI) for angina pectoris across groups of TyG index and TyG-WHtR.

| **Angina Pectoris** | | | | | | | | | | | | |
| --- | --- | --- | --- | --- | --- | --- | --- | --- | --- | --- | --- | --- |
|  | crude | | | | model1 | | | | model2 | | | |
|  | **OR**^1^ | **95% CI**^1^ | **p-value** | **p trend**^2^ | **OR**^1^ | **95% CI**^1^ | **p-value** | **p trend**^2^ | **OR**^1^ | **95% CI**^1^ | **p-value** | **p trend**^2^ |
| TyG |  |  |  | **<0.001** |  |  |  | **<0.001** |  |  |  | 0.078 |
| Q1 | — | — |  |  | — | — |  |  | — | — |  |  |
| Q2 | 2.09 | 1.36, 3.21 | <0.001 |  | 2.10 | 1.37, 3.22 | <0.001 |  | 2.37 | 1.16, 4.85 | **0.019** |  |
| Q3 | 2.38 | 1.54, 3.69 | <0.001 |  | 2.24 | 1.42, 3.54 | <0.001 |  | 3.01 | 1.50, 6.04 | **0.002** |  |
| Q4 | 2.92 | 1.73, 4.93 | <0.001 |  | 2.91 | 1.70, 4.97 | <0.001 |  | 2.93 | 1.23, 7.00 | **0.016** |  |
| TyG-WHtR |  |  |  | **<0.001** |  |  |  | **<0.001** |  |  |  | **0.013** |
| Q1 | — | — |  |  | — | — |  |  | — | — |  |  |
| Q2 | 1.78 | 1.06, 2.99 | 0.029 |  | 1.70 | 1.00, 2.88 | 0.048 |  | 2.02 | 0.97, 4.18 | 0.059 |  |
| Q3 | 1.31 | 0.78, 2.18 | 0.3 |  | 1.27 | 0.75, 2.15 | 0.4 |  | 1.68 | 0.77, 3.67 | 0.2 |  |
| Q4 | 1.66 | 0.97, 2.85 | 0.064 |  | 1.95 | 1.10, 3.48 | 0.024 |  | 2.61 | 1.20, 5.67 | **0.016** |  |
| ^1^HR = Hazard Ratio, CI = Confidence Interval  ^2^Tests for trends based on the variables containing the median values for each quartile.  crude was unadjusted, model 1 was adjusted for age, gender and race, model 2 was adjusted for age, gender, race, smoke, education, PIR, systolic blood pressure, total cholesterol, family history of heart disease, diabetes. | | | | | | | | | | | | |

**Supplementary Table 9.** Stratified analyses of the associations between TyG and All-cause mortality

| Total Mortality | | | | | | | |
| --- | --- | --- | --- | --- | --- | --- | --- |
|  |  | TyG | | | | p trend | p for interaction |
|  |  | Q1(Ref) | Q2(HR,95%CI) | Q3(HR,95%CI) | Q4(HR,95%CI) |  |  |
| Age | <60 | 1.00 | 1.57(0.88, 2.78) | 1.02(0.57, 1.82) | 1.46(0.82, 2.61) | 0.43 | 0.34 |
|  | >=60 | 1.00 | 1.02(0.80, 1.32) | 0.87(0.67, 1.12) | 1.07(0.82, 1.41) | 0.83 |  |
| Gender | female | 1.00 | 1.00(0.64, 1.56) | 0.89(0.58, 1.39) | 1.07(0.66, 1.72) | 0.99 | 0.92 |
|  | male | 1.00 | 1.15(0.88, 1.51) | 0.92(0.70, 1.21) | 1.13(0.85, 1.51) | 0.98 |  |
| Family poverty income ratio | ≤1.3 | 1.00 | 1.29(0.95, 1.75) | 0.89(0.65, 1.22) | 1.07(0.78, 1.48) | 0.97 | 0.36 |
|  | 1.31-3.5 | 1.00 | 0.86(0.66, 1.12) | 0.87(0.67, 1.13) | 0.94(0.71, 1.25) | 0.82 |  |
|  | >3.5 | 1.00 | 0.91(0.63, 1.31) | 0.74(0.50, 1.10) | 1.01(0.68, 1.50) | 0.37 |  |
| Race | Mexican America | 1.00 | 0.74(0.44, 1.23) | 0.65(0.39, 1.08) | 0.79(0.48, 1.30) | 0.59 | 0.79 |
|  | Other Hispainc | 1.00 | 0.59(0.23, 1.50) | 0.64(0.23, 1.78) | 1.26(0.50, 3.18) | 0.31 |  |
|  | Non-Hispanic white | 1.00 | 0.97(0.78, 1.22) | 0.83(0.67, 1.04) | 0.91(0.72, 1.15) | 0.13 |  |
|  | Non-Hispanic black | 1.00 | 1.30(0.90, 1.87) | 1.15(0.76, 1.76) | 1.57(0.99, 2.49) | 0.07 |  |
|  | Other | 1.00 | 0.55(0.19, 1.57) | 0.52(0.15, 1.75) | 0.51(0.16, 1.66) | 0.37 |  |
| Smoke | Never | 1.00 | 0.54(0.14, 2.07) | **0.22(0.05, 0.87) *** | 0.29(0.06, 1.39) | 0.10 | 0.73 |
|  | Former | 1.00 | 1.03(0.64, 1.66) | 0.80(0.49, 1.30) | 0.84(0.50, 1.41) | 0.37 |  |
|  | Now | 1.00 | 1.16(0.89, 1.52) | 0.94(0.71, 1.23) | 1.22(0.92, 1.63) | 0.63 |  |
| Drink | Never | 1.00 | 0.94(0.74, 1.20) | **0.76(0.60, 0.96) **** | 0.87(0.67, 1.12) | 0.21 | 0.85 |
|  | Mild-Moderate | 1.00 | 1.04(0.80, 1.34) | 0.99(0.76, 1.30) | 1.09(0.83, 1.45) | 0.94 |  |
| Education | Less than high school | 1.00 | 0.99(0.75, 1.30) | 0.76(0.58, 1.01) | 0.87(0.65, 1.17) | 0.33 | 0.31 |
|  | High school or equivalent | 1.00 | 0.91(0.62, 1.35) | 1.06(0.74, 1.53) | 1.31(0.89, 1.91) | 0.08 |  |
|  | College or above | 1.00 | 1.01(0.77, 1.33) | 0.84(0.62, 1.13) | 0.89(0.65, 1.22) | 0.11 |  |
| Systolic blood pressure | <130 | 1.00 | 1.08(0.85, 1.37) | 0.78(0.60, 1.01) | 0.99(0.75, 1.30) | 0.62 | 0.07 |
|  | >=130 | 1.00 | 0.92(0.71, 1.18) | 0.90 (0.70, 1.15) | 1.03(0.80, 1.33) | 0.92 |  |
| HBA1C | <6.5 | 1.00 | 1.05(0.87, 1.26) | 0.90 (0.74, 1.09) | 0.97(0.78, 1.22) | 0.66 | 0.68 |
|  | >=6.5 | 1.00 | 0.97(0.58, 1.60) | 0.92 (0.58, 1.45) | 1.09(0.70, 1.70) | 0.98 |  |
| Diabetes | No | 1.00 | 1.01(0.82, 1.24) | 0.89 (0.71, 1.11) | 0.94(0.71, 1.24) | 0.60 | 0.95 |
|  | Yes | 1.00 | 1.03(0.75, 1.41) | 0.86 (0.63, 1.16) | 1.02(0.76, 1.37) | 0.72 |  |

*p < 0.05, **p < 0.01, ***p < 0.001

**Supplementary Table 10**. Stratified analyses of the associations between TyG and CVD Mortality

| CVD Mortality | | | | | | | |
| --- | --- | --- | --- | --- | --- | --- | --- |
|  |  | TyG | | | | p trend | p for interaction |
|  |  | Q1(Ref) | Q2(HR,95%CI) | Q3(HR,95%CI) | Q4(HR,95%CI) |  |  |
| Age | <60 | 1.00 | 2.74(0.92, 8.19) | 2.06(0.69, 6.10) | 2.46(0.81, 7.49) | 0.25 | 0.64 |
|  | >=60 | 1.00 | 1.24(0.77, 2.00) | 1.16(0.73, 1.85) | 1.56(0.94, 2.59) | 0.10 |  |
| Gender | female | 1.00 | 1.11(0.50, 2.45) | 0.95(0.43, 2.10) | 0.88(0.36, 2.16) | 0.68 | 0.42 |
|  | male | 1.00 | 1.64(0.97, 2.75) | 1.53(0.91, 2.55) | **2.07(1.21, 3.55) **** | **0.01** |  |
| Family poverty  income ratio | ≤1.3 | 1.00 | 1.30(0.74, 2.28) | 0.85(0.48, 1.49) | 1.22(0.69, 2.17) | 0.58 | 0.45 |
|  | 1.31-3.5 | 1.00 | 1.00(0.64, 1.54) | 1.03(0.67, 1.59) | 1.06(0.66, 1.72) | 0.76 |  |
|  | >3.5 | 1.00 | 1.43(0.66, 3.07) | 1.58(0.74, 3.38) | **2.42(1.15, 5.07) *** | **0.01** |  |
| Race | Mexican America | 1.00 | 0.75(0.28, 2.04) | 0.92(0.37, 2.30) | 1.22(0.49, 3.02) | 0.36 | 0.51 |
|  | Other Hispainc | 1.00 | 0.40(0.10, 1.63) | 0.46(0.10, 2.16) | 0.92(0.20, 4.21) | 0.92 |  |
|  | Non-Hispanic white | 1.00 | 1.03(0.68, 1.56) | 0.96(0.64, 1.42) | 1.06(0.70, 1.61) | 0.82 |  |
|  | Non-Hispanic black | 1.00 | **2.10(1.12, 3.94) *** | 1.60(0.78, 3.29) | **2.74(1.29, 5.83) **** | **0.01** |  |
|  | Other | 1.00 | 1.32(0.12, 14.8) | 0.37(0.02, 7.28) | 1.56(0.12, 20.5) | 0.71 |  |
| Education | Less than high school | 1.00 | 0.80(0.48, 1.32) | 0.65(0.40, 1.05) | 0.82(0.49, 1.38) | 0.63 | 0.64 |
|  | High school or equivalent | 1.00 | 1.60(0.78, 3.28) | 1.84(0.93, 3.64) | **2.29(1.14, 4.59) *** | **0.02** |  |
|  | College or above | 1.00 | 1.37(0.83, 2.26) | 1.27(0.76, 2.15) | 1.36(0.79, 2.35) | 0.37 |  |
| Smoke | Never | 1.00 | 0.35(0.22, 2.00) | 0.25(0.09, 1.85) | 0.29(0.10, 1.26) | 0.22 | 0.11 |
|  | Former | 1.00 | 0.83(0.37, 1.88) | 0.54(0.22, 1.29) | 0.58(0.24, 1.42) | 0.19 |  |
|  | Now | 1.00 | **2.00(1.16, 3.45) *** | **1.72(1.01, 2.95) *** | 2.25(1.27, 3.98) | **0.02** |  |
| Drink | Never | 1.00 | 0.95(0.61, 1.47) | 0.82(0.54, 1.25) | 1.06(0.67, 1.66) | 0.75 | 0.61 |
|  | Mild-Moderate | 1.00 | 1.40(0.88, 2.21) | 1.40(0.88, 2.23) | 1.55(0.96, 2.49) | 0.09 |  |
| Systolic blood pressure | <130 | 1.00 | 1.21(0.79, 1.86) | 0.83(0.52, 1.31) | 1.10(0.68, 1.77) | 0.88 | 0.10 |
|  | >=130 | 1.00 | 1.09(0.69, 1.73) | 1.19(0.77, 1.85) | 1.52(0.97, 2.40) | **0.04** |  |
| HBA1C | <6.5 | 1.00 | 1.21(0.87, 1.69) | 1.10(0.78, 1.54) | 1.13(0.77, 1.67) | 0.57 | 0.67 |
|  | >=6.5 | 1.00 | 1.01(0.37, 2.74) | 1.13(0.48, 2.67) | 1.71(0.74, 3.92) | **0.04** |  |
| Diabetes | No | 1.00 | 0.97(0.54, 1.73) | 0.95(0.56, 1.60) | 1.20(0.72, 2.00) | 0.26 | 0.70 |
|  | Yes | 1.00 | 1.29(0.88, 1.87) | 1.14(0.77, 1.71) | 1.28(0.80, 2.04) | 0.33 |  |

*p < 0.05, **p < 0.01, ***p < 0.001

**Supplementary Table 11.** Stratified analyses of the associations between TyG and Total- CVD

| Total- CVD | | | | | | | |
| --- | --- | --- | --- | --- | --- | --- | --- |
|  |  | TyG | | | | p trend | P for interaction |
|  |  | Q1(Ref) | Q2(OR,95%CI) | Q3(OR,95%CI) | Q4(OR,95%CI) |  |  |
| Age | <60 | 1.00 | **2.08(1.06, 4.25) *** | **2.46(1.25,5.07) *** | **2.94(1.49, 6.09) **** | **0.01** | **0.02** |
|  | >=60 | 1.00 | 1.14(0.78, 1.65) | 1.15(0.79, 1.68) | **1.57(1.05, 2.36) *** | 0.16 |  |
| Gender | female | 1.00 | **2.05(1.12, 3.83) *** | 1.23(0.66, 2.34) | **2.24(1.16, 4.45) *** | 0.07 | 0.13 |
|  | male | 1.00 | 1.10(0.75, 1.61) | 1.46(1.00, 2.15) | **1.72(1.16, 2.57) **** | 0.05 |  |
| Family poverty  income ratio | ≤1.3 | 1.00 | 1.19(0.78, 1.82) | 1.01(0.66, 1.55) | 1.24 (0.79, 1.94) | 0.56 | 0.15 |
|  | 1.31-3.5 | 1.00 | 0.97(0.66, 1.43) | 1.24(0.84, 1.83) | **1.70(1.12, 2.58) *** | **0.01** |  |
|  | >3.5 | 1.00 | 1.42(0.86, 2.36) | **2.32(1.40, 3.89) ***** | **2.90(1.69, 5.02) ***** | **0.01** |  |
| Race | Mexican America | 1.00 | 1.03(0.47, 2.35) | 1.15(0.53, 2.63) | 1.00(0.45, 2.31) | **<0.001** | 0.90 |
|  | Other Hispainc | 1.00 | 1.13(0.42, 3.13) | 1.06(0.39, 2.99) | 1.54(0.57, 3.58) | 0.35 |  |
|  | Non-Hispanic white | 1.00 | 1.07(0.77, 1.50) | 1.24(0.89, 1.72) | **1.78(1.26, 2.53) **** | **0.02** |  |
|  | Non-Hispanic black | 1.00 | 1.34(0.79, 2.23) | **2.06(1.19, 3.5) **** | **2.64(1.43, 4.86) **** | **<0.001** |  |
|  | Other | 1.00 | 0.78(0.24, 2.59) | 0.57(0.16, 2.04) | 0.93(0.26, 3.51) | 0.61 |  |
| Education | Less than high school | 1.00 | 1.38(0.89, 2.16) | 1.31(0.84, 2.06) | **1.74(1.10, 2.78) *** | 0.05 | 0.80 |
|  | High school or equivalent | 1.00 | 1.04(0.61, 1.78) | 1.52(0.92, 2.53) | 1.71(1.00, 2.96) | 0.05 |  |
|  | College or above | 1.00 | 1.07(0.74, 1.54) | 1.31(0.90, 1.91) | **1.69(1.13, 2.54) **** | 0.15 |  |
| Smoke | Never | 1.00 | 0.83(0.22, 2.01) | 0.40(0.09, 1.77) | 0.55(0.36, 2.16) | 0.09 | 0.56 |
|  | Former | 1.00 | 1.35(0.71, 2.62) | 1.29(0.66, 2.55) | **2.08(1.07, 4.16) *** | 0.07 |  |
|  | Now | 1.00 | 1.26(0.87, 1.85) | 1.33(0.91, 1.96) | **1.75(1.17, 2.62) **** | 0.07 |  |
| Drink | Never | 1.00 | 0.94(0.66, 1.32) | 1.14(0.81, 1.60) | 1.15(0.80, 1.65) | 0.50 | 0.30 |
|  | Mild-Moderate | 1.00 | 1.43(1.00, 2.05) | **1.57(1.08, 2.28) *** | **2.65(1.81, 3.91) ***** | **<0.001** |  |
| Systolic blood pressure | <130 | 1.00 | 1.18(0.85, 1.64) | 1.34(0.95, 1.90) | **2.28(1.58, 3.29) ***** | **<0.001** | 0.20 |
|  | >=130 | 1.00 | 1.14(0.78, 1.67) | 1.30(0.91, 1.88)) | 1.41(0.96, 2.09) | 0.40 |  |
| HBA1C | <6.5 | 1.00 | 1.33(1.02, 1.75) | **1.49(1.12, 1.98) **** | **2.07(1.50, 2.84) ***** | **<0.001** | 0.35 |
|  | >=6.5 | 1.00 | 0.78(0.41, 1.47) | 1.14(0.66, 1.99) | 1.21(0.72, 2.10) | 0.79 |  |
| Diabetes | No | 1.00 | **1.52(1.10, 2.10) *** | **1.91(1.36, 2.68) ***** | **2.50(1.68, 3.73) ***** | **<0.001** | **0.04** |
|  | Yes | 1.00 | 0.78(0.52, 1.17) | 0.85(0.58, 1.24) | 1.10(0.76, 1.60) | 0.50 |  |

*p < 0.05, **p < 0.01, ***p < 0.001

**Supplementary Table 12.** Stratified analyses of the associations between TyG and Congestive Heart Failure

| Congestive Heart Failure | | | | | | | |
| --- | --- | --- | --- | --- | --- | --- | --- |
|  |  | TyG | | | | p trend | p for interaction |
|  |  | Q1(Ref) | Q2(OR,95%CI) | Q3(OR,95%CI) | Q4(OR,95%CI) |  |  |
| Age | <60 | 1.00 | 2.60(0.84, 9.87) | 2.68(0.86, 10.2) | 2.88(0.93, 11.1) | 0.18 | **0.04** |
|  | >=60 | 1.00 | 0.58(0.32, 1.03) | 0.87(0.50, 1.51) | 1.36 0.78, 2.40) | 0.86 |  |
| Gender | female | 1.00 | 0.82(0.32, 2.06) | 0.44(0.15, 1.19) | 1.96 0.79, 5.12) | 0.13 | **0.04** |
|  | male | 1.00 | 1.54(0.82, 2.36) | 1.53(0.88, 1.64) | **2.01(1.44, 3.56) *** | **0.03** |  |
| Family poverty  income ratio | ≤1.3 | 1.00 | 1.50(0.84, 2.71) | 1.08(0.58, 2.00) | 1.40(0.76, 2.64) | 0.56 | 0.43 |
|  | 1.31-3.5 | 1.00 | 0.73(0.42, 1.26) | 0.99(0.58, 1.68) | 1.47(0.86, 2.5) | 0.11 |  |
|  | >3.5 | 1.00 | 0.76(0.29, 1.98) | 1.48(0.62, 3.63) | 1.54(0.60, 4.05) | 0.22 |  |
| Race | Mexican America | 1.00 | 2.80(0.86, 12.6) | 1.49(0.41, 7.10) | 1.59(0.45, 7.57) |  | 0.80 |
|  | Other Hispainc | 1.00 | 0.65(0.16, 2.55) | 0.35(0.07, 1.55) | 1.01(0.27, 3.94) | 0.93 |  |
|  | Non-Hispanic white | 1.00 | 0.74(0.44, 1.26) | 1.14(0.71, 1.86) | 1.60(0.98, 2.65) | 0.97 |  |
|  | Non-Hispanic black | 1.00 | 0.99(0.49, 1.96) | 1.14(0.53, 2.36) | 1.16(0.49, 2.64) | 0.94 |  |
|  | Other | 1.00 | 7.24(0.55, 231) | 1.75(0.05, 76.4) | 12.0(0.70, 492) | 0.68 |  |
| Education | Less than high school | 1.00 | 1.15(0.65, 2.07) | 0.83(0.45, 1.53) | 0.95(0.52, 1.78) | 0.67 | 0.67 |
|  | High school or equivalent | 1.00 | 0.95(0.44, 2.02) | 1.30(0.68, 2.79) | 1.95(0.95, 4.12) | 0.06 |  |
|  | College or above | 1.00 | 0.81(.43, 1.50) | 1.26(0.69, 2.33) | **1.92(1.01, 3.66) *** | 0.78 |  |
| Smoke | Never | 1.00 | 0.69(0.22, 1.52) | 0.25(0.09, 2.05) | 0.19(0.10, 1.76) | 0.22 | 0.76 |
|  | Former | 1.00 | 1.61(0.55, 5.09) | 1.84(0.62, 5.92) | 2.85(0.96, 9.39) | 0.14 |  |
|  | Now | 1.00 | 0.68(0.38, 1.22) | 0.91(0.52, 1.60) | 1.31(0.75, 2.31) | 0.96 |  |
| Drink | Never | 1.00 | 0.92(0.56, 1.51) | 1.20(0.75, 1.94) | 1.48(0.91, 2.44) | 0.11 | 0.25 |
|  | Mild-Moderate | 1.00 | 1.24(0.79, 1.95) | 1.30(0.81, 2.09) | **2.10(1.30, 3.43) **** | **0.01** |  |
| Systolic blood pressure | <130 | 1.00 | 0.95(0.59, 1.55) | 1.23(0.75, 2.01) | **1.78(1.06, 2.99) *** | 0.05 | 0.93 |
|  | >=130 | 1.00 | 1.09(0.63, 1.91) | 1.00(0.58, 1.74) | 1.33(0.77, 2.35) | 0.57 |  |
| HBA1C | <6.5 | 1.00 | 1.15(0.77, 1.74) | 1.51(0.99, 2.30) | **1.71(1.05, 2.76) *** | **0.03** | 0.69 |
|  | >=6.5 | 1.00 | 0.77(0.34, 1.71) | 0.57(0.27, 1.19) | 1.02(0.53, 2.06) | 0.35 |  |
| Diabetes | No | 1.00 | 1.11(0.66, 1.87) | **1.85(1.12, 3.10) *** | **2.13(1.14, 3.93) *** | **0.01** | 0.08 |
|  | Yes | 1.00 | 0.83(0.50, 1.40) | 0.62(0.37, 1.04) | 1.01(0.63, 1.64) | 0.44 |  |

*p < 0.05, **p < 0.01, ***p < 0.001

**Supplementary Table 13.** Stratified analyses of the associations between TyG and Coronary Heart Disease

| Coronary Heart Disease | | | | | | | |
| --- | --- | --- | --- | --- | --- | --- | --- |
|  |  | TyG | | | | p trend | p for interaction |
|  |  | Q1(Ref) | Q2(OR,95%CI) | Q3(OR,95%CI) | Q4(OR,95%CI) |  |  |
| Age | <60 | 1.00 | 2.0(0.70, 6.61) | **3.30(1.19, 10.7) *** | **4.22(1.55, 13.7) **** | **0.01** | 0.07 |
|  | >=60 | 1.00 | 1.20(0.76, 1.93) | 1.07(0.66, 1.73) | 1.64(1.00, 2.73) | 0.05 |  |
| Gender | female | 1.00 | 1.38(0.55, 3.70) | 0.81(0.30, 2.26) | 1.80(0.69, 5.05) | 0.32 | 0.43 |
|  | male | 1.00 | 1.23(0.77, 1.99) | 1.51(0.93, 2.46) | **1.97(1.21, 3.26) **** | **0.01** |  |
| Family poverty  income ratio | ≤1.3 | 1.00 | 0.81(0.44, 1.49) | **0.49(0.25, 0.96) *** | 1.11(0.61, 2.06) | 0.73 | 0.10 |
|  | 1.31-3.5 | 1.00 | 1.09(0.64, 1.87) | 1.40(0.83, 2.37) | **2.01(1.16, 3.52) *** | **0.02** |  |
|  | >3.5 | 1.00 | 1.38(0.75, 2.57) | **2.49(1.36, 4.65) **** | **2.20(1.14, 4.31) *** | **0.01** |  |
| Race | Mexican America | 1.00 | 0.62(0.21, 1.85) | 0.71(0.24, 2.15) | 1.02(0.37, 3.05) | 0.70 | 0.25 |
|  | Other Hispainc | 1.00 | 1.77(0.43, 9.10) | 0.98(0.20, 5.45) | 1.71(0.38, 9.39) | 0.59 |  |
|  | Non-Hispanic white | 1.00 | 1.06(0.70, 1.60) | 1.14(0.76, 1.72) | **1.61(1.05, 2.47) *** | **0.03** |  |
|  | Non-Hispanic black | 1.00 | 1.31(0.47, 3.45) | **4.07(1.65, 10.2) **** | **5.78(2.17, 15.6) ***** | **<0.001** |  |
|  | Other | 1.00 | 0.51(0.12, 2.14) | 0.46(0.09, 2.14) | 0.76(0.17, 3.47) | 0.58 |  |
| Education | Less than high school | 1.00 | 1.05(0.57, 1.96) | 0.99(0.54, 1.85) | 1.80(0.99, 3.37) | 0.07 | 0.57 |
|  | High school or equivalent | 1.00 | 1.13(0.54, 2.39) | 1.25(0.61, 2.60) | 1.67(0.81, 3.55) | 0.17 |  |
|  | College or above | 1.00 | 1.12 (0.70, 1.80) | 1.56 (0.96, 2.54) | **1.73 (1.04, 2.92) *** | 0.05 |  |
| Smoke | Never | 1.00 | 0.84(0.35, 2.10) | 0.30(0.18,1.76) | 0.94(0.20, 1.37) | 0.22 | 0.20 |
|  | Former | 1.00 | 0.81 (0.33, 1.98) | 0.59(0.22, 1.55) | 1.43(0.59, 3.60) | 0.52 |  |
|  | Now | 1.00 | 1.42 (0.87, 2.35) | 1.51(0.92, 2.53) | **2.18(1.31, 3.68) **** | **<0.001** |  |
| Drink | Never | 1.00 | 0.92(0.56, 1.51) | 1.20(0.75, 1.94) | 1.48(0.91, 2.44) | 0.11 | 0.25 |
|  | Mild-Moderate | 1.00 | 1.24(0.79, 1.95) | 1.30(0.81, 2.09) | **2.10(1.30, 3.43) **** | **0.01** |  |
| Systolic blood pressure | <130 | 1.00 | 1.14(0.74, 1.77) | 1.28(0.82, 2.01) | **2.30(1.44, 3.70) ***** | **<0.001** | 0.51 |
|  | >=130 | 1.00 | 1.03(0.61, 1.75) | 1.18(0.71, 1.97) | 1.42(0.85, 2.40) | 0.14 |  |
| HBA1C | <6.5 | 1.00 | 1.22(0.85, 1.76) | 1.32(0.90, 1.94) | **2.00(1.32, 3.04) ***** | **0.01** | 0.79 |
|  | >=6.5 | 1.00 | 0.86(0.37, 2.02) | 1.20(0.59, 2.57) | 1.35(0.68, 2.83) | 0.16 |  |
| Diabetes | No | 1.00 | 1.51(0.98, 2.36) | **1.98(1.26, 3.14) **** | **2.94(1.73, 4.98) ***** | **<0.001** | 0.40 |
|  | Yes | 1.00 | 0.72(0.43, 1.22) | 0.74(0.45, 1.23) | 1.04(0.65, 1.70) | 0.20 |  |

*p < 0.05, **p < 0.01, ***p < 0.001

**Supplementary Table 14.** Stratified analyses of the associations between TyG and Angina pectoris

| Angina pectoris | | | | | | | |
| --- | --- | --- | --- | --- | --- | --- | --- |
|  |  | TyG | | | |  |  |
|  |  | Q1(Ref) | Q2(OR,95%CI) | Q3(OR,95%CI) | Q4(OR,95%CI) | p trend | p for interaction |
| Age | <60 | 1.00 | **4.29(1.34, 19.1) *** | 3.02(0.87, 14.0) | **4.79(1.46, 21.8) *** | 0.06 | 0.08 |
|  | >=60 | 1.00 | 1.32(0.75, 2.36) | 1.66(0.95, 2.95) | 1.25(0.67, 2.38) | 0.37 |  |
| Gender | female | 1.00 | **5.61(1.77, 25.0) **** | **4.35(1.35, 19.6) *** | **4.06(1.14, 19.4) *** | 0.23 | 0.19 |
|  | male | 1.00 | 1.19(0.67, 2.14) | 1.50(0.84, 2.71) | 1.46(0.79, 2.71) | 0.19 |  |
| Family poverty income ratio | ≤1.3 | 1.00 | 1.14(0.58, 2.28) | 1.42(0.73, 2.80) | 1.50(0.76, 3.04) | 0.24 | 0.48 |
|  | 1.31-3.5 | 1.00 | 1.58(0.87, 2.94) | 1.47(0.80, 2.79 | **1.92(1.02, 3.73) *** | 0.10 |  |
|  | >3.5 | 1.00 | 2.19(0.98, 5.27) | 3.00(1.33, 7.28) * | **3.71(1.60, 9.25) **** | **0.01** |  |
| Race | Mexican America | 1.00 | 0.77(0.27, 2.38) | 0.43(0.13, 1.46) | 0.48(0.16, 1.55) | 0.16 | 0.47 |
|  | Other Hispainc | 1.00 | 1.06(0.20, 6.31) | 0.80(0.14, 4.95) | 1.15(0.23, 6.84) | 0.78 |  |
|  | Non-Hispanic white | 1.00 | 1.34(0.80, 2.27) | 1.62(0.99, 2.72) | **2.08(1.24, 3.56) **** | **0.01** |  |
|  | Non-Hispanic black | 1.00 | **2.77(1.13, 7.04) *** | **4.89(1.98, 12.7) ***** | **4.09(1.36, 12.2) *** | **<0.001** |  |
|  | Other | 1.00 | 13.6(1.29, 381) | 3.02(0.09, 113) | **21.8(1.52, 77.9) *** | 0.28 |  |
| Education | Less than high school | 1.00 | 1.28(0.62, 2.73) | 1.70(0.86, 3.55) | 2.06(1.01, 4.42) | 0.05 | 0.09 |
|  | High school or equivalent | 1.00 | 1.29(0.52, 3.26) | **3.20(1.47, 7.51) **** | **2.70(1.16, 6.67) *** | **0.02** |  |
|  | College or above | 1.00 | **1.77(1.03, 3.11) *** | 1.07(0.57, 2.01) | 1.68(0.91, 3.17) | 0.33 |  |
| Smoke | Never | 1.00 | 0.85(0.22, 2.20) | 0.77(0.09, 2.86) | 0.20(0.12, 1.51) | 0.22 | 0.22 |
|  | Former | 1.00 | 2.15(0.86, 5.88) | 1.36(0.49, 3.97) | 2.03(0.75, 5.95) | 0.67 |  |
|  | Now | 1.00 | 1.46(0.80, 2.72) | **1.90(1.05, 3.53) *** | 1.64(0.87, 3.18) | 0.13 |  |
| Drink | Never | 1.00 | 1.24(0.73, 2.15) | 1.32(0.78, 2.27) | 1.48(0.86, 2.62) | 0.22 | 0.27 |
|  | Mild-Moderate | 1.00 | **2.08(1.17, 3.81) *** | **2.37(1.31, 4.41) **** | **3.06(1.65, 5.82) ***** | **<0.001** |  |
| Systolic blood pressure | <130 | 1.00 | **2.14(1.28, 3.67) **** | **2.03(1.18, 3.55) *** | **2.99(1.71, 5.33) ***** | **<0.001** | 0.36 |
|  | >=130 | 1.00 | 1.01(0.54, 1.89) | 1.37(0.77, 2.48) | 1.41(0.77, 2.65) | 0.21 |  |
| HBA1C | <6.5 | 1.00 | **1.70(1.11, 2.65) *** | **1.69(1.07, 2.69) *** | **2.44(1.49, 4.01) ***** | **<0.001** | 0.38 |
|  | >=6.5 | 1.00 | 1.31(0.53, 3.38) | 1.58(0.72, 3.76) | 1.47(0.68, 3.48) | 0.41 |  |
| Diabetes | No | 1.00 | **2.06(1.23, 3.49) **** | **2.21(1.30, 3.84) **** | **2.88(1.55, 5.35) ***** | **<0.001** | 0.23 |
|  | Yes | 1.00 | 1.06(0.58, 1.99) | 1.14(0.64, 2.10) | 1.39(0.80, 2.51) | 0.15 |  |

**Supplementary Table 15.** Stratified analyses of the associations between TyG-WHtR and All-cause Mortality

| All-cause Mortality | | | | | | | |
| --- | --- | --- | --- | --- | --- | --- | --- |
|  |  | TyG-WHtR | | | |  |  |
|  |  | Q1(Ref) | Q2(HR,95%CI) | Q3(HR,95%CI) | Q4(HR,95%CI) | p trend | p for interaction |
| Age | <60 | 1.00 | 0.78(0.45, 1.36) | 1.0(0.60, 1.64) | 1.26(0.78, 2.03) | 0.20 | **<0.001** |
|  | >=60 | 1.00 | 0.91(0.71, 1.16) | 0.82(0.64, 1.06) | 1.14(0.88, 1.50) | 0.32 |  |
| Gender | female | 1.00 | 0.90(0.55, 1.49) | 0.90(0.56, 1.46) | 1.18(0.73, 1.91) | 0.22 | 0.78 |
|  | male | 1.00 | 0.88(0.69, 1.13) | 0.89(0.68, 1.16) | **1.36(1.03, 1.81) *** | **0.04** |  |
| Family poverty income ratio | ≤1.3 | 1.00 | 0.86(0.63, 1.17) | 0.74(0.54, 1.02) | 1.08(0.79, 1.47) | 0.40 | 0.21 |
|  | 1.31-3.5 | 1.00 | 0.84(0.65, 1.09) | 0.98(0.76, 1.27) | 1.04(0.78, 1.38) | 0.44 |  |
|  | >3.5 | 1.00 | 0.94(0.66, 1.34) | 0.93(0.63, 1.38) | 1.33(0.91, 1.95) | 0.17 |  |
| Race | Mexican America | 1.00 | 1.25(0.76, 2.05) | 0.81(0.48, 1.36) | 1.11(0.66, 1.86) | 0.93 | 0.40 |
|  | Other Hispainc | 1.00 | 1.11(0.47, 2.60) | 1.13(0.44, 2.86) | 2.16(0.83, 5.62) | 0.11 |  |
|  | Non-Hispanic white | 1.00 | 0.85(0.68, 1.06) | 0.99(0.79, 1.24) | 1.03(0.82, 1.31) | 0.39 |  |
|  | Non-Hispanic black | 1.00 | 0.70(0.47, 1.04) | 0.73(0.49, 1.11) | 1.18(0.76, 1.83) | 0.52 |  |
|  | Other | 1.00 | 0.90(0.31, 2.60) | 0.29(0.08, 1.07) | **4.38(1.46,13.2) **** | 0.09 |  |
| Smoke | Former | 1.00 | 1.43(0.43, 4.74) | 0.28(0.04, 1.79) | 5.54(0.91, 33.8) | 0.17 | 0.99 |
|  | Now | 1.00 | 1.03(0.66, 1.61) | 1.08(0.69, 1.70) | 1.27(0.80, 2.02) | 0.29 |  |
|  | Never | 1.00 | 0.88(0.67, 1.15) | 0.87(0.66, 1.14) | **1.34(1.00, 1.78) *** | **0.03** |  |
| Drink | Never | 1.00 | 0.89(0.70, 1.12) | 0.86(0.68, 1.10) | 1.05(0.82, 1.35) | 0.50 | 0.92 |
|  | Mild-Moderate | 1.00 | 0.92(0.72, 1.19) | 0.96(0.74, 1.25) | 1.22(0.92, 1.61) | 0.13 |  |
| Education | Less than high school | 1.00 | 0.80(0.61, 1.06) | 0.76(0.57, 1.02) | 1.00(0.75, 1.33) | 0.66 | 0.93 |
|  | High school or equivalent | 1.00 | 1.07(0.75, 1.51) | 1.13(0.78, 1.62) | 1.39(0.95, 2.02) | 0.07 |  |
|  | College or above | 1.00 | 0.86(0.65, 1.14) | 0.93(0.70, 1.24) | 1.08(0.79, 1.48) | 0.52 |  |
| Systolic blood pressure | <130 | 1.00 | **0.78(0.61, 1.00) *** | 0.84(0.65, 1.08) | 1.09(0.83, 1.42) | 0.34 | 0.71 |
|  | >=130 | 1.00 | 0.99(0.78, 1.25) | 0.98(0.76, 1.25) | 1.19(0.92, 1.54) | 0.13 |  |
| HBA1C | <6.5 | 1.00 | 0.88(0.73, 1.07) | 0.94(0.77, 1.15) | 1.09(0.87, 1.36) | 0.40 | 0.36 |
|  | >=6.5 | 1.00 | 1.01(0.67, 1.51) | 0.93(0.63, 1.37) | 1.28(0.88, 1.85) | 0.06 |  |
| Diabetes | No | 1.00 | 0.85(0.69, 1.06) | 0.97(0.77, 1.22) | 1.13(0.87, 1.47) | 0.32 | 0.18 |
|  | Yes | 1.00 | 0.96(0.72, 1.28) | 0.86(0.65, 1.15) | 1.10(0.83, 1.46) | 0.29 |  |

*p < 0.05, **p < 0.01, ***p < 0.001

**Supplementary Table 16.** Stratified analyses of the associations between TyG-WHtR and CVD Mortality

| CVD Mortality | | | | | | | |
| --- | --- | --- | --- | --- | --- | --- | --- |
|  |  | TyG-WHtR | | | | p trend | p for interaction |
|  |  | Q1(Ref) | Q2(HR,95%CI) | Q3(HR,95%CI) | Q4(HR,95%CI) |  |  |
| Age | <60 | 1.00 | 2.67(0.93, 7.65) | 2.59(0.89, 7.54) | **2.89(1.01, 8.28) *** | 0.09 | **0.03** |
|  | >=60 | 1.00 | 1.17(0.74, 1.86) | 1.07(0.66, 1.71) | **1.77(1.08, 2.90) *** | **0.02** |  |
| Gender | female | 1.00 | **4.60(1.28, 16.6) *** | 2.55(0.70, 9.26) | **5.84(1.63, 20.9) **** | **0.01** | 0.27 |
|  | male | 1.00 | 1.12(0.70, 1.78) | 1.32(0.82, 2.12) | **1.78(1.06, 2.99) *** | **0.02** |  |
| Family poverty income ratio | ≤1.3 | 1.00 | 0.88(0.49, 1.57) | 0.83(0.46, 1.49) | 1.32(0.74, 2.35) | 0.19 | 0.81 |
|  | 1.31-3.5 | 1.00 | 0.98(0.63, 1.53) | 1.03(0.65, 1.62) | 1.44(0.89, 2.33) | 0.10 |  |
|  | >3.5 | 1.00 | 1.42(0.75, 2.71) | 1.35(0.67, 2.71) | **2.68(1.40, 5.13 **** | **<0.001** |  |
| Race | Mexican America | 1.00 | 2.04(0.79, 5.24) | 1.53(0.58, 4.02) | 2.13(0.80, 5.70) | 0.25 | 0.41 |
|  | Other Hispainc | 1.00 | 0.72(0.15, 3.46) | 1.01(0.17, 5.83) | 2.25(0.43, 11.9) | 0.16 |  |
|  | Non-Hispanic white | 1.00 | 1.03(0.68, 1.54) | 1.18(0.79, 1.78) | **1.52(1.00, 2.30) *** | **0.02** |  |
|  | Non-Hispanic black | 1.00 | 0.93(0.49, 1.79) | 0.73(0.35, 1.51) | 1.85(0.90, 3.79) | 0.16 |  |
|  | Other | 1.00 | 0.62(0.06, 6.67) | 0.25(0.02, 3.68) | 6.39(0.76, 53.5) | 0.09 |  |
| Education | Less than high school | 1.00 | 0.85(0.50, 1.43) | 0.82(0.49, 1.40) | 1.34(0.78, 2.29) | 0.12 | 0.17 |
|  | High school or equivalent | 1.00 | 1.19(0.64, 2.21) | 1.47(0.80, 2.68) | 1.45(0.75, 2.83) | 0.23 |  |
|  | College or above | 1.00 | 1.35(0.83, 2.21) | 1.11(0.65, 1.90) | **2.27(1.36, 3.80) **** | **<0.001** |  |
| Smoke | Never | 1.00 | 0.33(0.27, 2.96) | 0.85(0.09, 1.32) | 0.23(0.42, 1.29) | 0.22 | 0.99 |
|  | Former | 1.00 | 1.42(0.64, 3.17) | 1.39(0.58, 3.30) | 1.48(0.61, 3.55) | 0.43 |  |
|  | Now | 1.00 | 1.50(0.86, 2.59) | 1.54(0.89, 2.66) | **2.64(1.49, 4.69) ***** | **<0.001** |  |
| Drink | Never | 1.00 | 0.99(0.64, 1.51) | 1.01(0.66, 1.56) | **1.63(1.05, 2.54) *** | **0.01** | 0.52 |
|  | Mild-Moderate | 1.00 | 1.32(0.85, 2.05) | 1.21(0.76, 1.93) | **1.79(1.12, 2.88) *** | **0.02** |  |
| Systolic blood pressure | <130 | 1.00 | 0.90(0.57, 1.42) | 1.04(0.65, 1.66) | **1.70(1.06, 2.72) *** | **0.01** | 0.57 |
|  | >=130 | 1.00 | 1.28(0.84, 1.96) | 1.18(0.76, 1.82) | **1.78(1.14, 2.77) *** | **0.01** |  |
| HBA1C | <6.5 | 1.00 | 1.15(0.82, 1.62) | 1.17(0.82, 1.68) | **1.70(1.16, 2.49) **** | **0.01** | 0.71 |
|  | >=6.5 | 1.00 | 1.02(0.50, 2.06) | 0.92(0.46, 1.84 | 1.61(0.84, 3.09) | **0.03** |  |
| Diabetes | No | 1.00 | 1.03(0.69, 1.52) | 1.29(0.87, 1.93) | **2.02(1.31, 3.13) **** | **<0.001** | 0.57 |
|  | Yes | 1.00 | 1.23(0.73, 2.06) | 0.93(0.55, 1.58) | 1.48(0.89, 2.46) | 0.11 |  |

*p < 0.05, **p < 0.01, ***p < 0.001

**Supplementary Table 17.** Stratified analyses of the associations between TyG-WHtR and Total-CVD

| Total-CVD | | | | | | | |
| --- | --- | --- | --- | --- | --- | --- | --- |
|  |  |  | | | | p trend | p for interaction |
|  |  | Q1(Ref) | Q2(OR,95%CI) | Q3(OR,95%CI) | Q4(OR,95%CI) |  |  |
| Age | <60 | 1.00 | 1.32(0.70, 2.50) | 1.68(0.94, 3.07) | **2.28(1.27, 4.20) **** | **<0.001** | 0.17 |
|  | >=60 | 1.00 | 0.97(0.67, 1.40) | 1.04(0.71, 1.52) | 1.26(0.84, 1.89) | 0.18 |  |
| Gender | female | 1.00 | 1.25(0.63, 2.58) | 0.99(0.51, 2.01) | 1.46(0.77, 2.89) | 0.31 | 0.57 |
|  | male | 1.00 | 0.98(0.69, 1.40) | 1.31(0.91, 1.88) | **1.54(1.04, 2.28) *** | **0.01** |  |
| Family poverty income ratio | ≤1.3 | 1.00 | 1.00(0.64, 1.56) | 0.88(0.56, 1.38) | 1.53(0.99, 2.37) | **0.04** | 0.08 |
|  | 1.31-3.5 | 1.00 | 0.93(0.63, 1.38) | 1.21(0.83, 1.78) | 1.29(0.86, 1.94) | 0.23 |  |
|  | >3.5 | 1.00 | 1.32(0.84, 2.07) | 1.45(0.90, 2.32) | **1.72(1.04, 2.85) *** | **0.03** |  |
| Race | Mexican America | 1.00 | 0.74(0.37, 1.51) | 0.70(0.34, 1.42) | 1.01(0.50, 2.06) | 0.79 | 0.39 |
|  | Other Hispainc | 1.00 | 1.34(0.42, 3.61) | 2.03(0.67, 7.02) | 3.16(1.05, 11.1) | **0.02** |  |
|  | Non-Hispanic white | 1.00 | 1.15(0.83, 1.60) | 1.23(0.88, 1.72) | **1.47(1.05, 2.06) *** | **0.04** |  |
|  | Non-Hispanic black | 1.00 | 0.74(0.43, 1.26) | 0.79(0.45, 1.36) | 1.28(0.73, 2.26) | 0.61 |  |
|  | Other | 1.00 | 2.42(0.78, 7.67) | 2.78(0.96, 8.36) | 3.06(0.86, 10.9) | 0.07 |  |
| Education | Less than high school | 1.00 | 1.08(0.69, 1.69) | 1.19(0.77, 1.86) | 1.52(0.97, 2.39) | 0.07 | 0.71 |
|  | High school or equivalent | 1.00 | 1.11(0.66, 1.88) | 1.30(0.76, 2.21) | 1.68(0.99, 2.88) | 0.11 |  |
|  | College or above | 1.00 | 1.02(0.71, 1.45) | 1.08 0.75, 1.56 | 1.37 0.94, 2.01 | 0.10 |  |
| Smoke | Never | 1.00 | 2.94(0.40, 25.6) | 2.29(0.11, 37.8) | 1.51(0.14, 18.2) | 0.97 | 0.89 |
|  | Former | 1.00 | 1.10(0.60, 2.03) | 1.57(0.85, 2.90) | 1.79(0.96, 3.37) | **0.04** |  |
|  | Now | 1.00 | 1.00(0.68, 1.47) | 1.10(0.76, 1.61) | 1.44(0.97, 2.15) | 0.06 |  |
| Drink | Never | 1.00 | 0.99(0.71, 1.40) | 0.84(0.59, 1.19 | 1.24(0.88, 1.77 | 0.41 | 0.16 |
|  | Mild-Moderate | 1.00 | 1.11(0.78, 1.58) | **1.66(1.18, 2.36) **** | **1.83(1.27, 2.65) ***** | **<0.001** |  |
| Systolic blood pressure | <130 | 1.00 | 1.24(0.89, 1.73) | **1.42(1.02, 1.99) **** | **1.83(1.29, 2.60) ***** | **<0.001** | 0.28 |
|  | >=130 | 1.00 | 0.86(0.60, 1.24) | 0.92(0.64, 1.34) | 1.23(0.85, 1.78) | 0.24 |  |
| HBA1C | <6.5 | 1.00 | 1.05(0.80, 1.39) | 1.27(0.96, 1.67) | 1.33(0.98, 1.82) | **0.02** | **0.01** |
|  | >=6.5 | 1.00 | 1.14(0.65, 2.02) | 0.99(0.58, 1.73) | 1.67(1.00, 2.85) | **0.01** |  |
| Diabetes | No | 1.00 | 0.92(0.66, 1.26) | 1.21(0.88, 1.68) | 1.34(0.92, 1.93) | 0.09 | 0.14 |
|  | Yes | 1.00 | 1.36(0.91, 2.03) | 1.22(0.83, 1.82) | **1.73)1.19, 2.55) **** | **0.01** |  |

*p < 0.05, **p < 0.01, ***p < 0.001

**Supplementary Table 18.** Stratified analyses of the associations between TyG-WHtR and Congestive Heart Failure

| Congestive Heart Failure | | | | | | | |
| --- | --- | --- | --- | --- | --- | --- | --- |
|  |  | TyG-WHtR | | | | p trend | p for interaction |
|  |  | Q1(Ref) | Q2(OR,95%CI) | Q3(OR,95%CI) | Q4(OR,95%CI) |  |  |
| Age | <60 | 1.00 | **5.38(1.30, 36.4) *** | **5.52(1.44, 36.3) *** | **13.3(3.67, 86.0) ***** | **<0.001** | **0.02** |
|  | >=60 | 1.00 | 0.92(0.51, 1.70) | 1.38(0.78, 2.51) | **1.98(1.09, 3.66) *** | **0.01** |  |
| Gender | female | 1.00 | 0.51(0.14, 1.80) | 1.19(0.45, 3.57) | 1.62(0.63, 4.75) | 0.07 | 0.40 |
|  | male | 1.00 | 1.49(0.92, 2.46) | 1.92(1.19, 3.15) | **3.25(2.00, 5.36) ***** | **<0.001** |  |
| Family poverty income ratio | ≤1.3 | 1.00 | 1.28(0.64, 2.64) | 1.54(0.78, 3.15) | **3.27(1.73, 6.50) ***** | **<0.001** | **0.08** |
|  | 1.31-3.5 | 1.00 | 0.86(0.50, 1.50) | 0.95(0.55, 1.64) | 1.62(0.95, 2.81) | 0.07 |  |
|  | >3.5 | 1.00 | 0.97(0.37, 2.54) | 1.94(0.81, 4.82) | 2.23(0.89, 5.76) | **0.04** |  |
| Race | Mexican America | 1.00 | 0.55(0.18, 1.65) | 0.68(0.24, 1.97) | 1.32(0.50, 3.68) | 0.31 | 0.79 |
|  | Other Hispainc | 1.00 | 1.44(0.24, 11.4) | 2.31(0.45, 17.7) | 3.63(0.72, 28.4) | 0.12 |  |
|  | Non-Hispanic white | 1.00 | 1.27(0.75, 2.22) | 1.45(0.85, 2.52) | **2.27(1.36, 3.91) ***** | **<0.001** |  |
|  | Non-Hispanic black | 1.00 | 0.76(0.33, 1.67) | 1.03(0.47, 2.25) | **2.34(1.10, 5.05) *** | **0.03** |  |
|  | Other | 1.00 | 0.66(0.02, 9.92) | 1.63(0.19, 15.7) | 4.37(0.46, 53.1) | 0.06 |  |
| Education | Less than high school | 1.00 | 0.89(0.46, 1.72) | 1.43(0.79, 2.67) | 1.73(0.94, 3.27) | **0.03** | **0.04** |
|  | High school or equivalent | 1.00 | 1.05(0.50, 2.24) | 1.31(0.63, 2.80) | 1.86(0.91, 3.95) | 0.08 |  |
|  | College or above | 1.00 | 1.10(0.58, 2.14) | 0.99(0.49, 1.98) | **3.12(1.69, 5.93) ***** | **<0.001** |  |
| Smoke | Never | 1.00 | 0.84(0.61, 2.00) | 0.43(0.09, 1.75) | 0.89(0.30, 1.06) | 0.22 | 0.76 |
|  | Former | 1.00 | 1.19(0.42, 3.40) | 2.12(0.79, 5.96) | **3.35(1.27, 9.47) *** | **0.01** |  |
|  | Now | 1.00 | 1.22(0.64, 2.39) | 1.59(0.86, 3.06) | **2.64(1.43, 5.08) **** | **<0.001** |  |
| Drink | Never | 1.00 | 0.91(0.54, 1.53) | 0.92(0.55, 1.56) | **1.76(1.08, 2.92) *** | **0.01** | 0.46 |
|  | Mild-Moderate | 1.00 | 1.14(0.63, 2.09) | **1.90(1.08, 3.39) *** | **2.82(1.60, 5.09) ***** | **<0.001** |  |
| Systolic blood pressure | <130 | 1.00 | 1.26(0.75, 2.14) | 1.58(0.94, 2.69) | **2.79(1.68, 4.72) ***** | **<0.001** | 0.29 |
|  | >=130 | 1.00 | 0.76(0.42, 1.38) | 1.03(0.59, 1.82) | 1.70(0.99, 2.97) | **0.01** |  |
| HBA1C | <6.5 | 1.00 | 1.07(0.69, 1.68) | **1.64(1.07, 2.53) *** | **2.13(1.36, 3.38) ***** | **<0.001** | **<0.001** |
|  | >=6.5 | 1.00 | 0.98(0.44, 2.27) | 0.80(0.36, 1.86) | **2.25(1.12, 4.86) *** | **<0.001** |  |
| Diabetes | No | 1.00 | 0.89(0.52, 1.51) | 1.40(0.84, 2.35) | **1.78(1.02, 3.11) *** | **0.02** | 0.38 |
|  | Yes | 1.00 | 1.20(0.67, 2.22) | 1.21(0.68, 2.21) | **2.45(1.44, 4.35) ***** | **<0.001** |  |

*p < 0.05, **p < 0.01, ***p < 0.001

**Supplementary Table 19.** Stratified analyses of the associations between TyG-WHtR and Coronary Heart Disease

| Coronary Heart Disease | | | | | | | |
| --- | --- | --- | --- | --- | --- | --- | --- |
|  |  | TyG-WHtR | | | | p trend | p for interaction |
|  |  | Q1(Ref) | Q2(OR,95%CI) | Q3(OR,95%CI) | Q4(OR,95%CI) |  |  |
| Age | <60 | 1.00 | 1.13(0.45, 2.78) | 1.54(0.67, 3.61) | **2.51(1.13, 5.84) *** | **0.01** | 0.68 |
|  | >=60 | 1.00 | 1.16(0.73, 1.87) | 1.38(0.86, 2.25) | 1.50(0.91, 2.51) | 0.07 |  |
| Gender | female | 1.00 | 1.06(0.37, 3.26) | 0.92(0.34, 2.78) | 1.00(0.38, 2.94) | 0.88 | 0.74 |
|  | male | 1.00 | 1.18(0.75, 1.86) | 1.56(0.99, 2.47) | **2.04(1.26, 3.32) **** | **<0.001** |  |
| Family poverty income ratio | ≤1.3 | 1.00 | 0.80(0.42, 1.53) | 0.87(0.46, 1.66) | 1.07(0.58, 2.01) | 0.57 | 0.91 |
|  | 1.31-3.5 | 1.00 | 1.22(0.73, 2.09) | 1.41(0.84, 2.40) | 1.59(0.92, 2.79) | 0.23 |  |
|  | >3.5 | 1.00 | 1.19(0.70, 2.03) | 1.12(0.62, 2.01) | 1.55(0.84, 2.84) | 0.14 |  |
| Race | Mexican America | 1.00 | 0.98(0.37, 2.72) | 0.91(0.33, 2.58) | 1.30(0.49, 3.68) | 0.57 | 0.78 |
|  | Other Hispainc | 1.00 | 0.61(0.10, 3.62) | 1.58(0.36, 8.46) | 1.68(0.37, 9.44) | 0.29 |  |
|  | Non-Hispanic white | 1.00 | 1.12(0.75, 1.67) | 1.12(0.75, 1.67) | 1.30(0.86, 1.97) | 0.27 |  |
|  | Non-Hispanic black | 1.00 | 0.78(0.31, 1.85) | 0.81(0.31, 2.00) | 1.88(0.75, 4.65) | 0.27 |  |
|  | Other | 1.00 | 1.82(0.48, 6.85) | 2.64(0.78, 9.37) | 1.43(0.26, 6.76) | 0.43 |  |
| Education | Less than high school | 1.00 | 1.27(0.70, 2.36) | 1.15(0.63, 2.16) | 1.49(0.81, 2.82) | 0.30 | 0.37 |
|  | High school or equivalent | 1.00 | 1.34(0.65, 2.84) | 1.30(0.62, 2.83) | 1.82(0.87, 3.92) | 0.26 |  |
|  | College or above | 1.00 | 0.90(0.57, 1.41) | 1.16(0.74, 1.83) | 1.19(0.73, 1.94) | 0.34 |  |
| Smoke | Never | 1.00 | 0.74(0.32, 1.85) | 0.44(0.10, 0.97) | 0.60(0.26, 1.55) | 0.22 | 0.45 |
|  | Former | 1.00 | 0.92(0.37, 2.26) | 1.36(0.56, 3.33) | 2.24(0.96, 5.45) | **0.03** |  |
|  | Now | 1.00 | 1.21(0.74, 1.99) | 1.48(0.91, 2.42) | **1.72(1.04, 2.89) *** | **0.02** |  |
| Drink | Never | 1.00 | 1.01(0.64, 1.61) | 0.78(0.48, 1.27) | 1.16(0.72, 1.87) | 0.78 | 0.30 |
|  | Mild-Moderate | 1.00 | 1.12(0.72, 1.76) | **1.68(1.08, 2.62) *** | **1.65(1.02, 2.67) *** | **0.02** |  |
| Systolic blood pressure | <130 | 1.00 | 1.33(0.88, 2.04) | 1.44(0.93, 2.22) | 1.46(0.92, 2.32) | 0.17 | 0.24 |
|  | >=130 | 1.00 | 0.80(0.49, 1.31) | 0.90(0.55, 1.48) | 1.29(0.79, 2.12) | 0.17 |  |
| HBA1C | <6.5 | 1.00 | 1.15(0.81, 1.65) | 1.29(0.89, 1.86) | 1.14(0.74, 1.74) | 0.53 | 0.12 |
|  | >=6.5 | 1.00 | 1.00(0.50, 2.08) | 1.05(0.53, 2.14) | 1.71(0.91, 3.37) | **0.02** |  |
| Diabetes | No | 1.00 | 0.99(0.65, 1.50) | 1.31(0.85, 2.01) | 1.03(0.59, 1.75) | 0.60 | 0.35 |
|  | Yes | 1.00 | 1.32(0.79, 2.25) | 1.20(0.72, 2.04) | **1.75(1.08, 2.93) *** | **0.02** |  |

*p < 0.05, **p < 0.01, ***p < 0.001

**Supplementary Table 20.** Stratified analyses of the associations between TyG-WHtR and Angina Pectoris

| Angina Pectoris | | | | | | | |
| --- | --- | --- | --- | --- | --- | --- | --- |
|  |  | TyG-WHtR | | | | p trend | p for interaction |
|  |  | Q1(Ref) | Q2(OR,95%CI) | Q3(OR,95%CI) | Q4(OR,95%CI) |  |  |
| Age | <60 | 1.00 | 1.58(0.57, 4.56) | 2.07(0.82, 5.68) | 2.26(0.89, 6.27) | 0.09 | 0.78 |
|  | >=60 | 1.00 | 1.27(0.73, 2.28) | 1.22(0.68, 2.25) | 1.72(0.94, 3.20) | 0.08 |  |
| Gender | female | 1.00 | 2.63(0.79, 11.9) | 2.22(0.68, 9.94) | 2.44(0.77, 10.8) | 0.51 | 0.44 |
|  | male | 1.00 | 1.14(0.66, 1.98) | 1.28(0.73, 2.28) | **1.92(1.08, 3.45) *** | **0.02** |  |
| Family poverty income ratio | ≤1.3 | 1.00 | 1.85(0.88, 4.20) | 2.03(0.96, 4.59) | 2.14(1.02, 4.85) | 0.13 | 0.88 |
|  | 1.31-3.5 | 1.00 | 1.28(0.70, 2.39) | 1.37(0.75, 2.55) | 1.52(0.81, 2.90) | 0.33 |  |
|  | >3.5 | 1.00 | 1.49(0.77, 2.91) | 1.24(0.59, 2.59) | 1.92(0.91, 4.05) | 0.12 |  |
| Race | Mexican America | 1.00 | 0.46(0.14, 1.46) | 0.69(0.24, 2.02) | 0.89(0.33, 2.60) | 0.73 | 0.08 |
|  | Other Hispainc | 1.00 | 0.54(0.31, 2.65) | 1.68(0.36,7.44) | 0.68(0.47, 6.34) | 0.19 |  |
|  | Non-Hispanic white | 1.00 | 1.45(0.90, 2.39) | 1.38(0.84, 2.31) | 1.5(0.97, 2.67) | 0.13 |  |
|  | Non-Hispanic black | 1.00 | 1.59(0.68, 3.86) | 1.05(0.39, 2.75) | 1.28(0.47, 3.47) | 0.82 |  |
|  | Other | 1.00 | 1.05(0.32, 5.43) | 2,04(0.97,8.33) | 1.23(0.26, 6.26) | 0.13 |  |
| Education | Less than high school | 1.00 | **2.54(1.11, 6.58) *** | **3.00(1.33, 7.67) *** | **4.19(1.85, 10.8) ***** | **<0.001** | 0.25 |
|  | High school or equivalent | 1.00 | 1.21(0.54, 2.81) | 1.64(0.75, 3.78) | 2.05(0.94, 4.73) | 0.08 |  |
|  | College or above | 1.00 | 1.35(0.81, 2.28) | 0.99 (0.56, 1.74) | 0.89(0.49, 1.63) | 0.43 |  |
| Smoke | Never | 1.00 | 0.54(0.78, 2.23) | 0.89(0.54, 2.13) | 0.20(0.10, 1.65) | 0.22 | 0.09 |
|  | Former | 1.00 | 2.27(0.89, 6.26) | 1.94(0.71, 5.60) | 2.27(0.85, 6.57) | 0.48 |  |
|  | Now | 1.00 | 1.31(0.71, 2.44) | 1.31(0.71, 2.46) | **2.02(1.10, 3.83) *** | **0.03** |  |
| Drink | Never | 1.00 | 1.78(1.01, 3.25) | **1.81(1.03, 3.30) *** | **1.90(1.07, 3.50) *** | 0.82 | 0.99 |
|  | Mild-Moderate | 1.00 | 1.31(0.78, 2.23) | **1.30(0.75, 2.26) *** | 1.60(0.92, 2.83) | 0.29 |  |
| Systolic blood pressure | <130 | 1.00 | **1.90(1.17, 3.15) *** | 1.66(0.99, 2.81) | **1.99(1.17, 3.42) *** | 0.05 | 0.11 |
|  | >=130 | 1.00 | 0.99(0.53, 1.85) | 1.27(0.71, 2.35) | 1.36(0.75, 2.52) | 0.28 |  |
| HBA1C | <6.5 | 1.00 | 1.45(0.95, 2.24) | 1.54(0.99, 2.39) | 1.37(0.83, 2.24) | 0.24 | **0.02** |
|  | >=6.5 | 1.00 | 2.08(0.85, 5.91) | 1.85(0.76, 5.18) | **2.61(1.13, 7.14) *** | 0.05 |  |
| Diabetes | No | 1.00 | 1.29(0.80, 2.08) | 1.39(0.84, 2.28) | 0.92(0.48, 1.69) | 0.98 | **0.01** |
|  | Yes | 1.00 | **2.13(1.09, 4.48) *** | 1.96(1.01, 4.13) | **2.83(1.49, 5.84) **** | **<0.001** |  |

*p < 0.05, **p < 0.01, ***p < 0.001
